# Supplementary material for: MOZ/ENL complex is a recruiting factor of leukemic AF10 fusion proteins
Source: Nat Commun. 2023 Apr 8;14:1979. doi: 10.1038/s41467-023-37712-5 (PMC10082848; doi:10.1038/s41467-023-37712-5)
Supplement: Supplementary file 1 — Supplementary info [file 41467_2023_37712_MOESM1_ESM.pdf]

Supplementary Information

MOZ/ENL complex is a recruiting factor of leukemic AF10 fusion proteins

Yosuke Komata, et al.

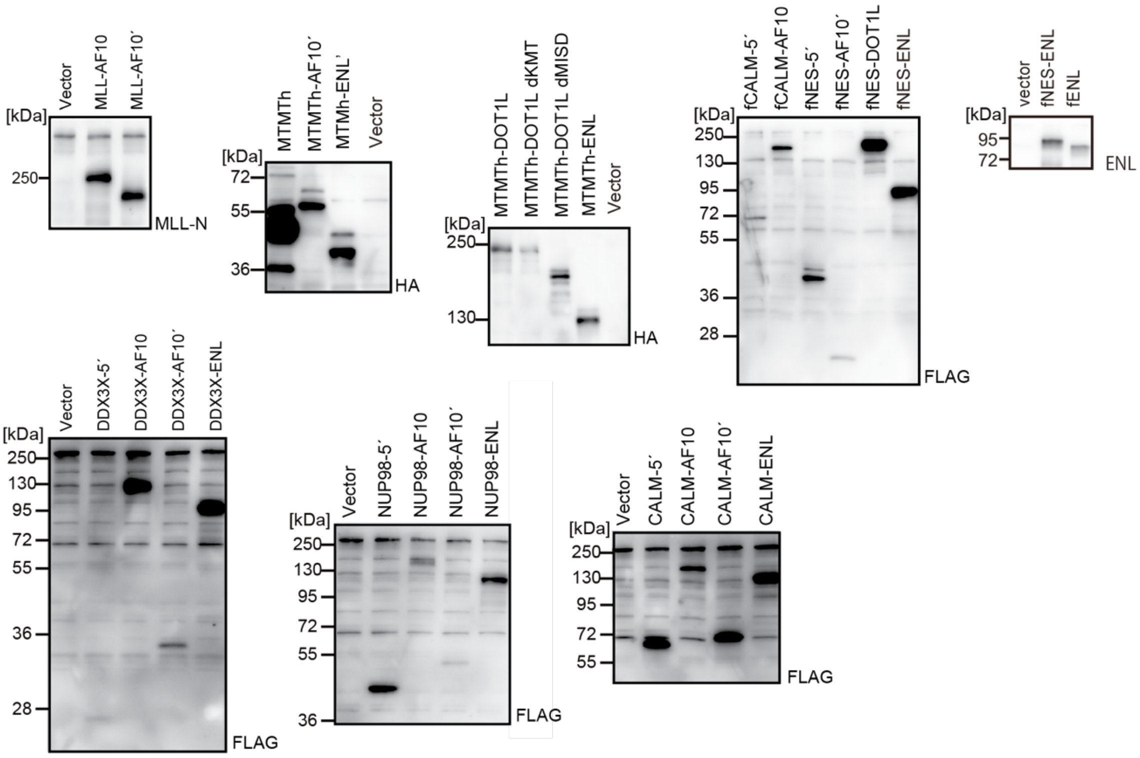

Supplementary Fig. 1 Profiles of AF10 fusions mentioned in Fig. 1. Protein expression of various AF10 fusion constructs. WB analysis of the whole-cell extract of virus-packaging cells with specific antibodies.

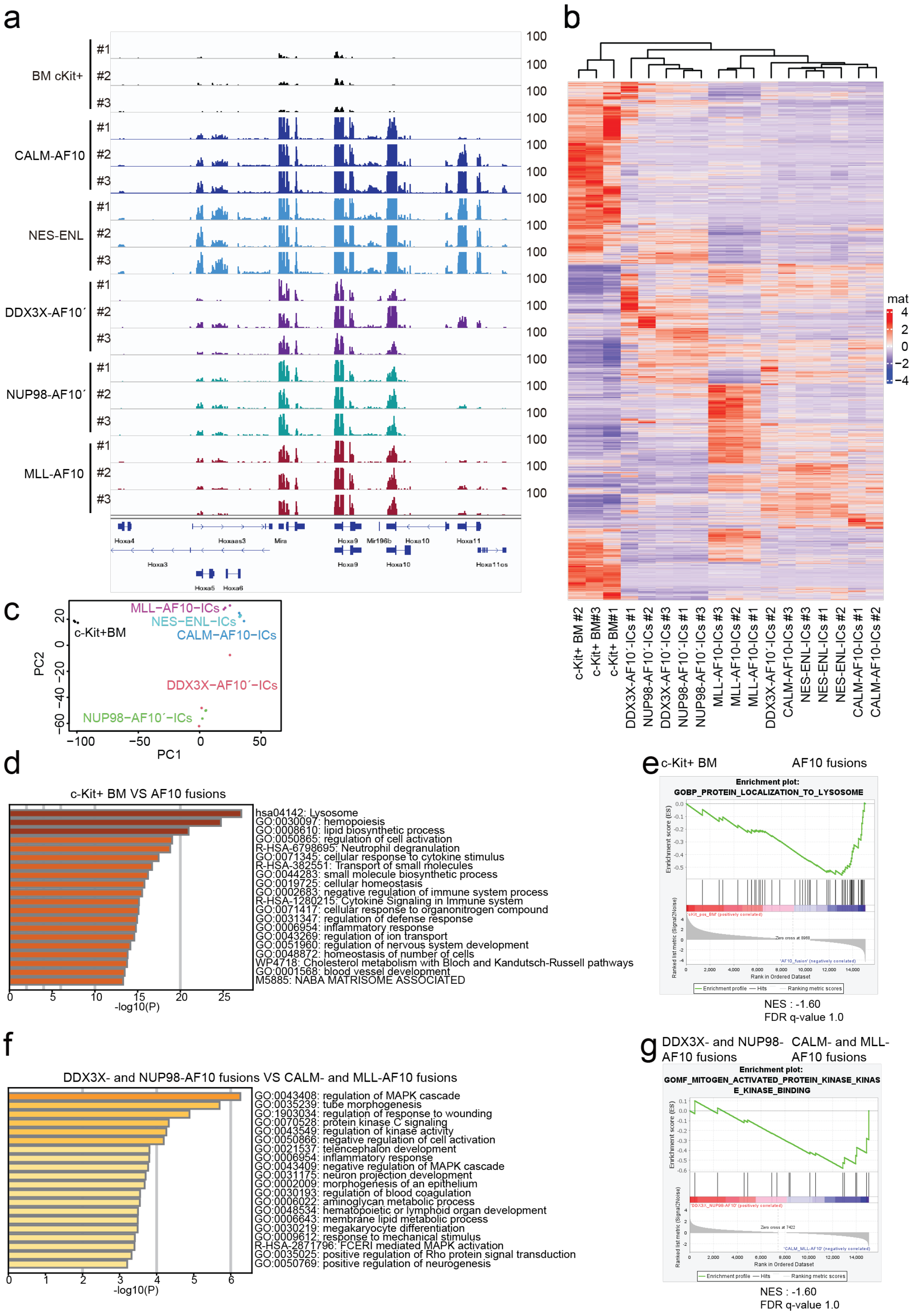
a
BM cKit+
CALM-AF10
NES-ENL
DDX3X-AF10´
NUP98-AF10´
MLL-AF10
#1
#2
#3
100
Hoxa4
Hoxaas3
Mira
Hoxa9
Mir196b
Hoxa10
Hoxa11
Hoxa3
Hoxa11os
Hoxa5
Hoxa6
b
mat
4
2
0
−2
−4
c-Kit+ BM #2
c-Kit+ BM#3
c-Kit+ BM#1
DDX3X-AF10´-ICs #1
NUP98-AF10´-ICs #2
DDX3X-AF10´-ICs #3
NUP98-AF10´-ICs #1
NUP98-AF10´-ICs #3
MLL-AF10-ICs #3
MLL-AF10-ICs #2
MLL-AF10-ICs #1
DDX3X-AF10´-ICs #2
CALM-AF10-ICs #3
NES-ENL-ICs #3
NES-ENL-ICs #1
NES-ENL-ICs #2
CALM-AF10-ICs #1
CALM-AF10-ICs #2
c
MLL−AF10−ICs
NES−ENL−ICs
CALM−AF10−ICs
c-Kit+BM
DDX3X−AF10´−ICs
NUP98−AF10´−ICs
PC2
PC1
d
c-Kit+ BM VS AF10 fusions
hsa04142: Lysosome
GO:0030097: hemopoiesis
GO:0008610: lipid biosynthetic process
GO:0050865: regulation of cell activation
R-HSA-6798695: Neutrophil degranulation
GO:0071345: cellular response to cytokine stimulus
R-HSA-382551: Transport of small molecules
GO:0044283: small molecule biosynthetic process
GO:0019725: cellular homeostasis
GO:0002683: negative regulation of immune system process
R-HSA-1280215: Cytokine Signaling in Immune system
GO:0071417: cellular response to organonitrogen compound
GO:0031347: regulation of defense response
GO:0006954: inflammatory response
GO:0043269: regulation of ion transport
GO:0051960: regulation of nervous system development
GO:0048872: homeostasis of number of cells
WP4718: Cholesterol metabolism with Bloch and Kandutsch-Russell pathways
GO:0001568: blood vessel development
M5885: NABA MATRISOME ASSOCIATED
-log10(P)
e
c-Kit+ BM
AF10 fusions
Enrichment plot:
GOBP_PROTEIN_LOCALIZATION_TO_LYSOSOME
Enrichment score (ES)
Ranked list metric (Signal2Noise)
Rank in Ordered Dataset
Enrichment profile — Hits — Ranking metric scores
NES : -1.60
FDR q-value 1.0
f
DDX3X- and NUP98-AF10 fusions VS CALM- and MLL-AF10 fusions
GO:0043408: regulation of MAPK cascade
GO:0035239: tube morphogenesis
GO:1903034: regulation of response to wounding
GO:0070528: protein kinase C signaling
GO:0043549: regulation of kinase activity
GO:0050866: negative regulation of cell activation
GO:0021537: telencephalon development
GO:0006954: inflammatory response
GO:0043409: negative regulation of MAPK cascade
GO:0031175: neuron projection development
GO:0002009: morphogenesis of an epithelium
GO:0030193: regulation of blood coagulation
GO:0006022: aminoglycan metabolic process
GO:0048534: hematopoietic or lymphoid organ development
GO:0006643: membrane lipid metabolic process
GO:0030219: megakaryocyte differentiation
GO:0009612: response to mechanical stimulus
R-HSA-2871796: FCERI mediated MAPK activation
GO:0035025: positive regulation of Rho protein signal transduction
GO:0050769: positive regulation of neurogenesis
-log10(P)
g
DDX3X- and NUP98-AF10 fusions
CALM- and MLL-AF10 fusions
Enrichment plot:
GOMF_MITOGEN_ACTIVATED_PROTEIN_KINASE_KINASE_KINASE_BINDING
Enrichment score (ES)
Ranked list metric (Signal2Noise)
Rank in Ordered Dataset
Enrichment profile — Hits — Ranking metric scores
NES : -1.60
FDR q-value 1.0

Supplementary Fig. 2 RNA expression profiles of AF10 fusion-immortalized cells mentioned in Fig. 1. a, RNA expression in various AF10 fusion-immortalized cells. RNA-seq analysis of the immortalized cells (ICs) for various AF10 fusions. Three independently established cell lines for each construct were analyzed using RNA-seq along with three different preparations of c-Kit-positive cells from healthy bone marrow (BM). **b**, Heatmap of RNA-seq profiles of AF10 fusion-ICs. A total of 8849 differentially expressed genes in AF10 fusion-ICs were clustered using the ComplexHeatmap R package. **c**, Principal component analysis of RNA-seq profiles of AF10 fusion-ICs using R. **d**, **e**, Pathways affected by the differentially expressed genes between C-Kit+ BM cells VS AF10 fusion ICs using Metascape (**d**) and GSEA (**e**). **f**, **g**, Pathways affected by the differentially expressed genes between DDX3X- and NUP98-AF10-ICs VS MLL- and CALM-AF10-ICs using Metascape (**f**) and GSEA (**g**).

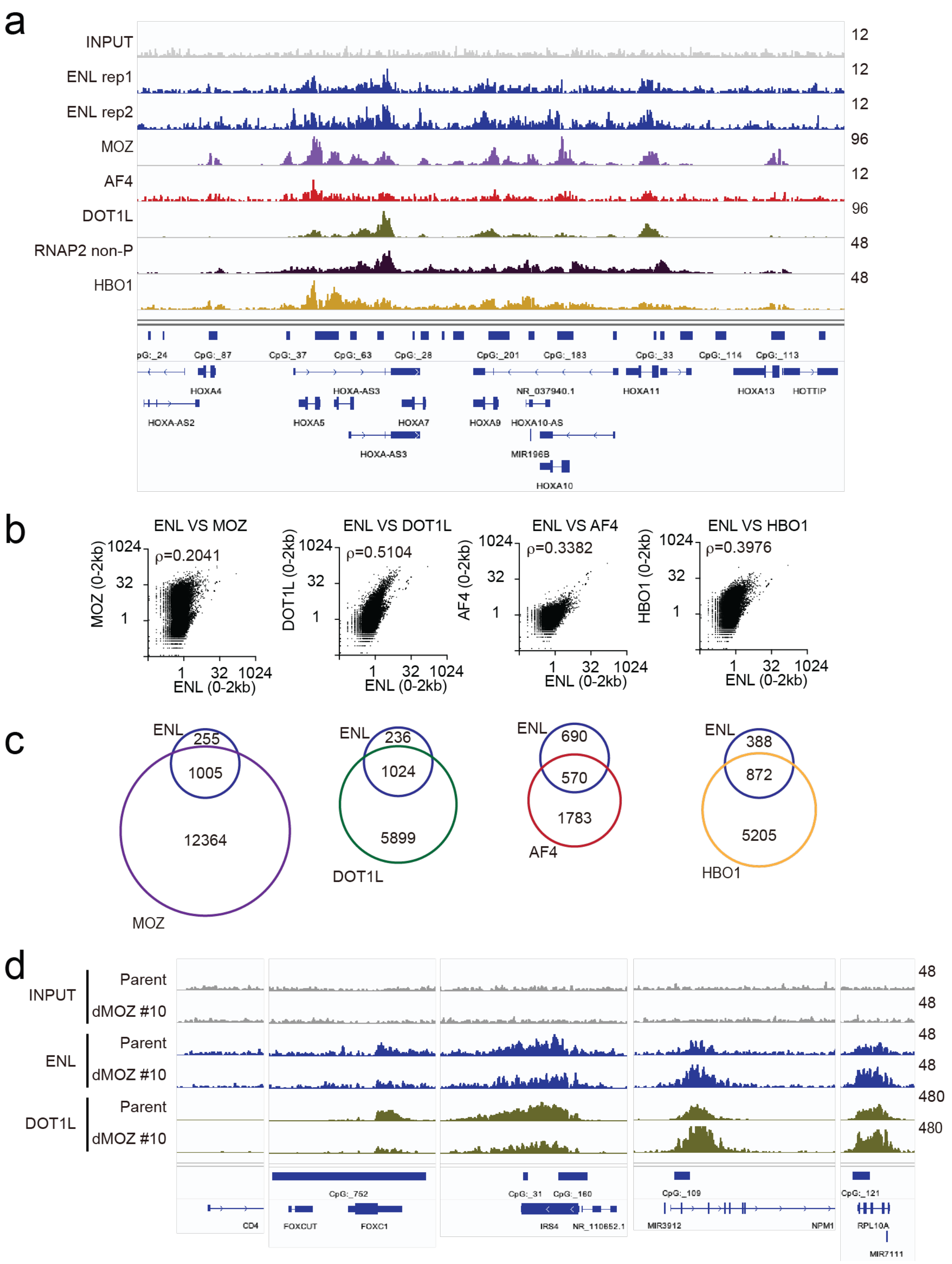

Supplementary Fig. 3 Genomic localization profiles of various components of ENL, MYST KATs, and other ENL binders mentioned in Fig. 2. a, ChIP-seq profiles of various ENL-associated factors at the *HOXA* locus. The data were visualized using the Integrative Genomics Viewer (Broad Institute). **b**, Correlations between ChIP signal intensities of ENL and its binding proteins. Relative occupation by multiple factors in all the TSSs. The ChIP-seq

tags of the indicated proteins (for all genes) clustered into a 2 kb bin (0 to +2 kb from the TSS) are presented as XY scatter plots with Spearman's rank correlation coefficient (ρ). **c**, Commonalities of target genes of ENL, MOZ, DOT1L, AF4, and HBO1. Genes whose relative ratio of ChIP/INPUT signals in 2kb bins of 0 to 2kb were larger than 2 were defined as target genes. Commonalities of each target genes were depicted using Venn diagrams. **d**, Representative ChIP-seq profiles of the genes where the presence of ENL and DOT1L are dependent on MOZ (e.g., FOXC1 and IRS4) or not (e.g., NPM1 and RPL10A).

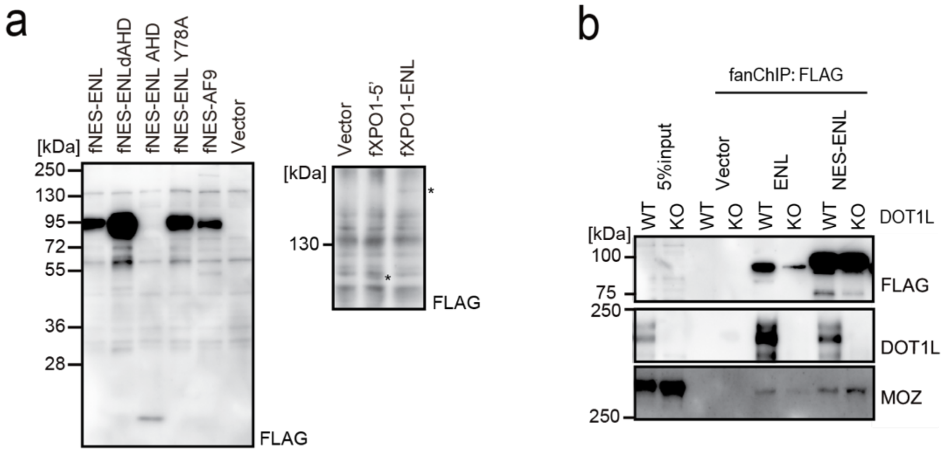

Supplementary Fig. 4 Profiles of NES-ENL fusion proteins mentioned in Fig. 3. a, Protein expression of various ENL constructs. WB of the whole cell extract of virus-packaging cells was performed using anti-FLAG antibody. * indicates the positions of exogenously expressed proteins **b**, ENL-MOZ interaction in the absence of DOT1L. IP-WB was performed on the chromatin fraction of HEK293T cells harboring endogenous DOT1L (WT) or deficient for DOT1L (KO) that are transiently expressing various ENL constructs. The co-precipitates were immunoblotted with anti-FLAG, -DOT1L, and -MOZ antibodies.

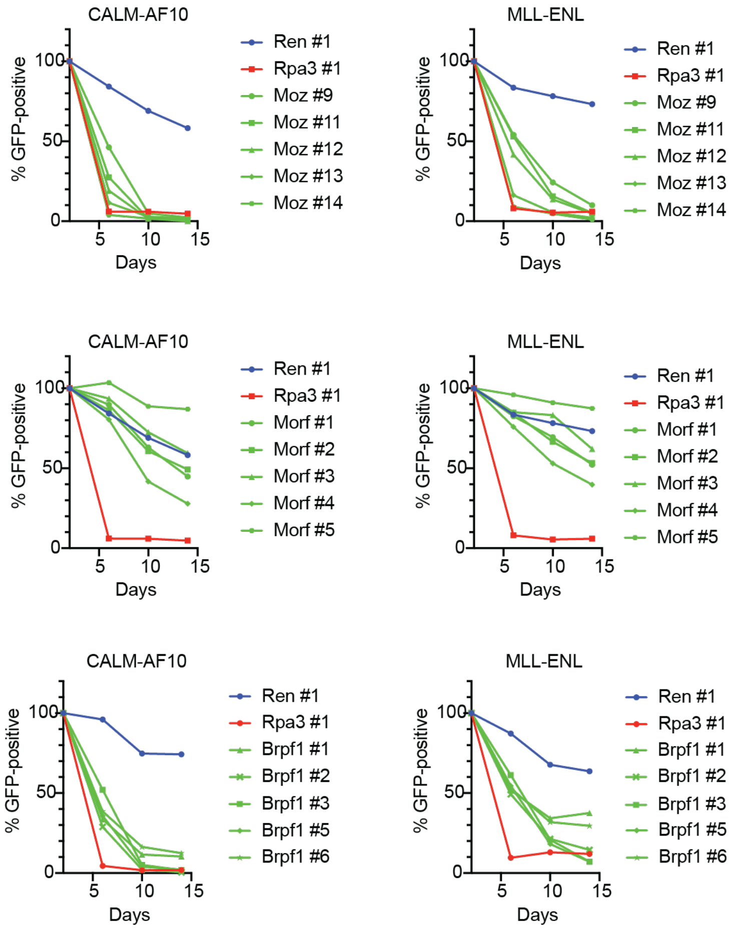

Supplementary Fig. 5 Dependencies on MOZ, MORF, and BRPF1 of CALM-AF10- and MLL-ENL-immortalized cells. a, Requirement of MOZ, MORF, and BRPF1 for myeloid progenitors immortalized by CALM-AF10 or MLL-ENL were analyzed using multiple different sgRNAs as in Fig.4.

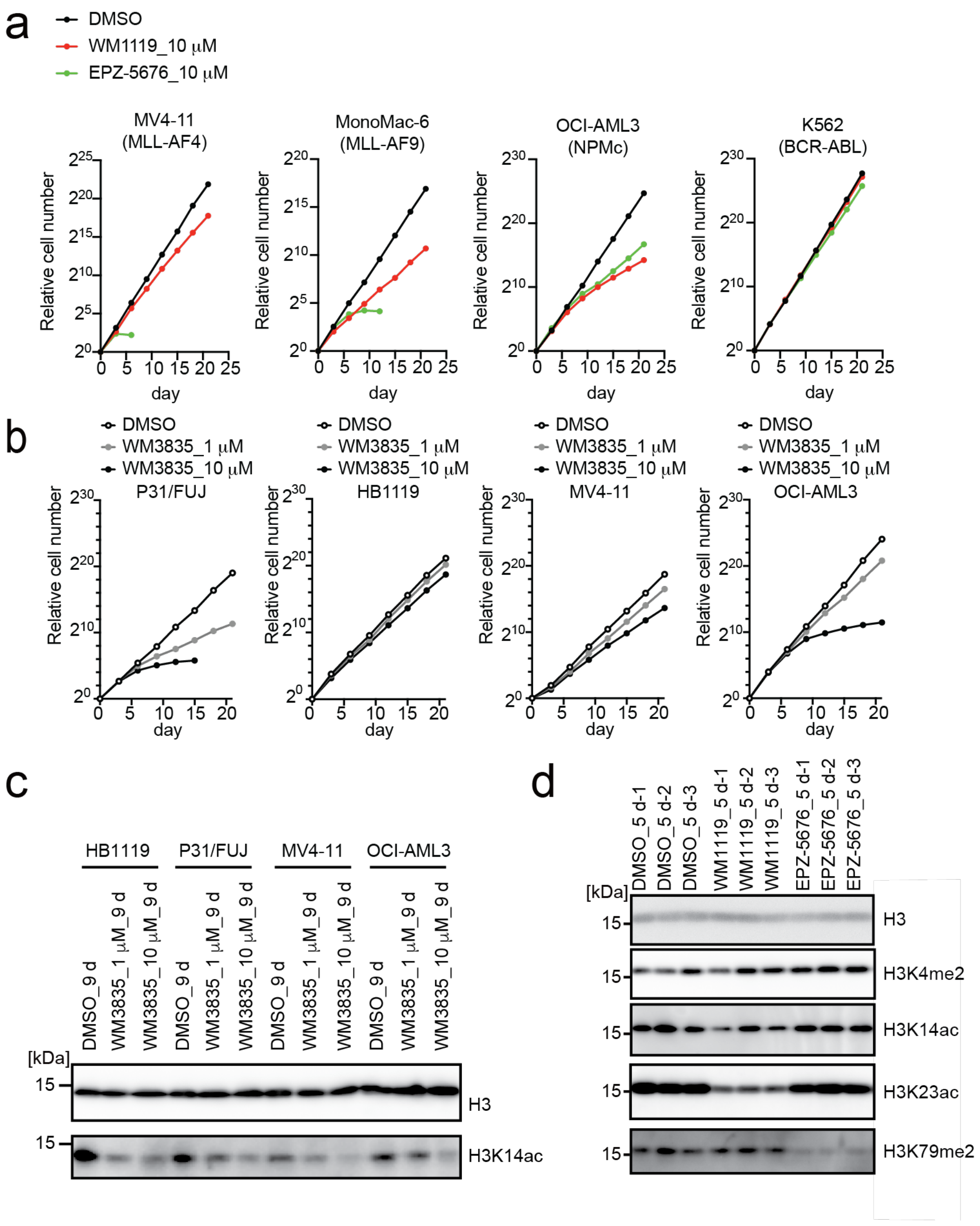

Supplementary Fig. 6 Effects of MOZ/MORF KAT and DOT1L KMT inhibitors on various cell lines mentioned in Fig. 5. a, Effects of MOZ/MORF KAT and DOT1L KMT inhibitors on the proliferation of various cell lines. The proliferation of the indicated cell lines was monitored for 21 days in the presence of 10 μM WM1119 or EPZ-5676, along with the DMSO control. **b**, Effects of a MOZ/MORF KAT inhibitor in the proliferation of various cell lines. The proliferation of the indicated cell lines in the presence of 1 or 10 μM WM3835 was monitored for 21 days along with the DMSO control. **c**, Histone acetylation of various leukemia cell lines treated with MOZ/MORF KAT inhibitor. WB of the whole cell extract of various leukemia cells 9 days after treatment with 1 or 10 μM WM3835 using antibodies specific to histone H3 and H3K14ac marks. **d**, Histone modifications in HEK293T cells after treatment with MOZ/MORF KAT and DOT1L KMT inhibitors. WB of the whole-cell extracts of the HEK293T

cells treated with 10 μM WM1119 or EPZ-5676 for 5 days (three biological replicates) using antibodies specific to each histone modification.

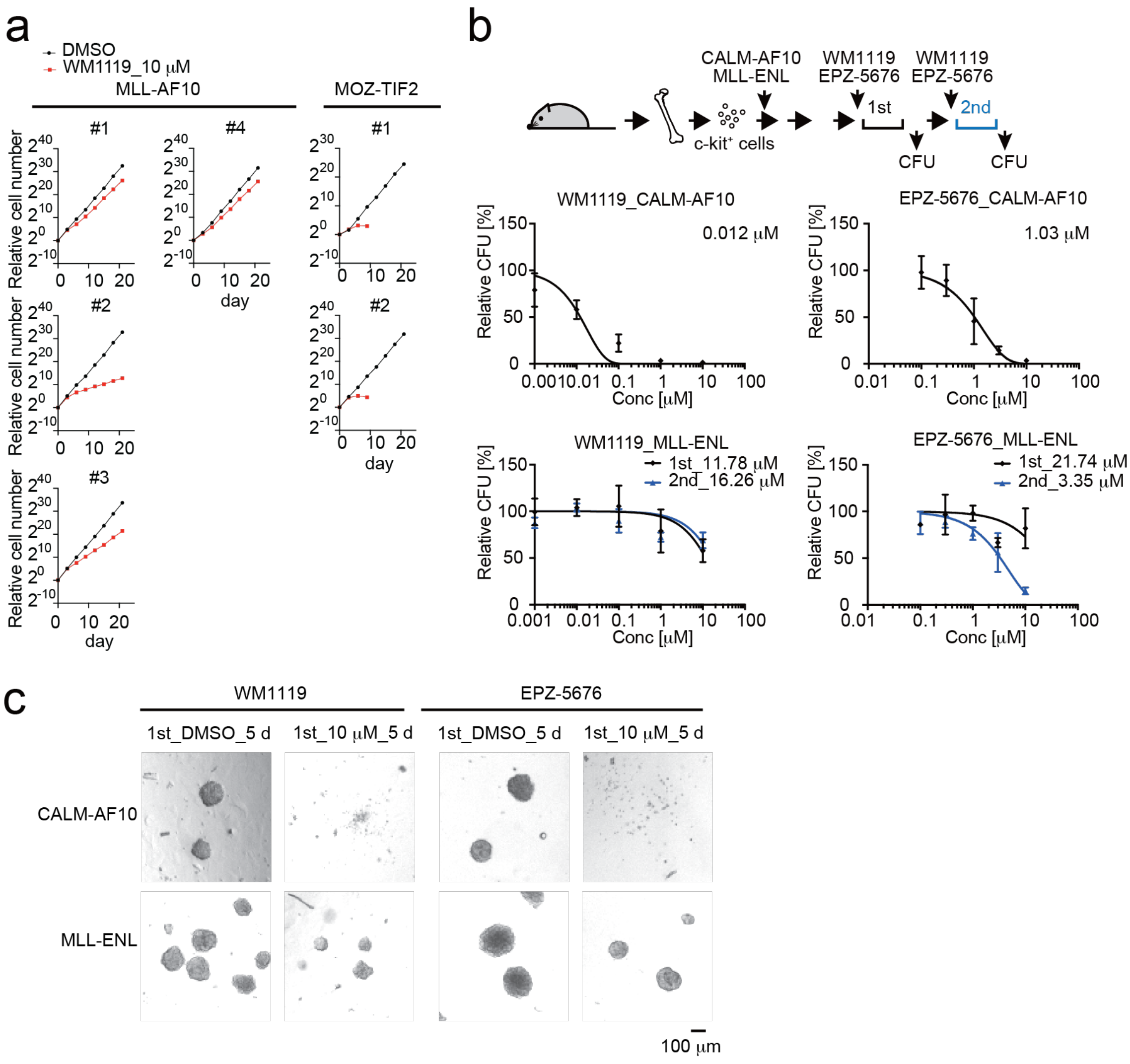

Supplementary Fig. 7 Effects of MOZ/MORF inhibition on murine immortalized cells mentioned in Fig. 6. a, Proliferation of MLL-AF10- and MOZ-TIF2-ICs in the presence of MOZ/MORF KAT inhibitors (10 μM WM-1119) was monitored for 21 days along with the vehicle control (DMSO) as in Fig. 6b. **b**, Effects of MOZ/MORF KAT and DOT1L KMT inhibition on the proliferation of CALM-AF10- and MLL-ENL-ICs. Cells were cultured in a semi-solid medium in the presence or absence of WM1119 or EPZ-5676 at the indicated range of doses and the resultant relative colony-forming activity was measured (mean ± SD of technical replicates, n=3). For MLL-ENL-ICs, the analysis was extended to the second passage. **c**, Representative images of colonies.

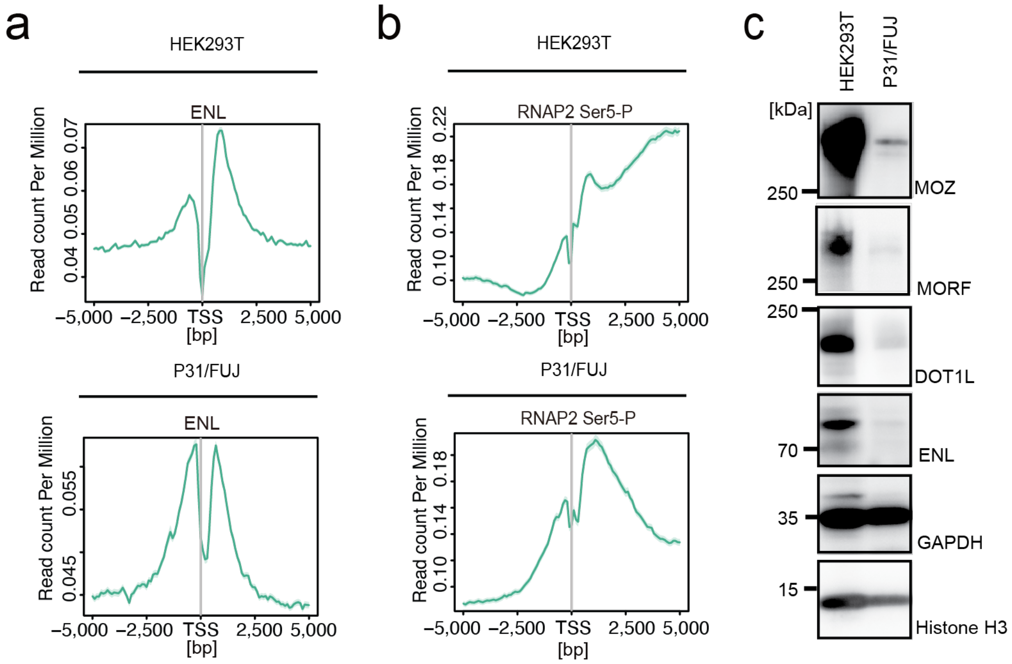

Supplementary Fig. 8 Effects of dual inhibition with MOZ/MORF KATs and DOT1L KMT inhibitors mentioned in Fig. 7. a, Average distribution of ENL near the TSSs. The ChIP signal distribution of ENL within the 5 kb range near the TSSs was shown using the ngsplot software[1]. **b**, Average distribution of RNA Polymerase II with its CTD phosphorylated on Ser5 of the heptapeptide repeats (RNAP2 Ser5-P) near the TSSs is shown as in **a**. **c**, Protein expression in HEK293T and P31/FUJ cells. The whole cell extracts of HEK293T and P31/FUJ cells were analyzed by WB using indicated antibodies.

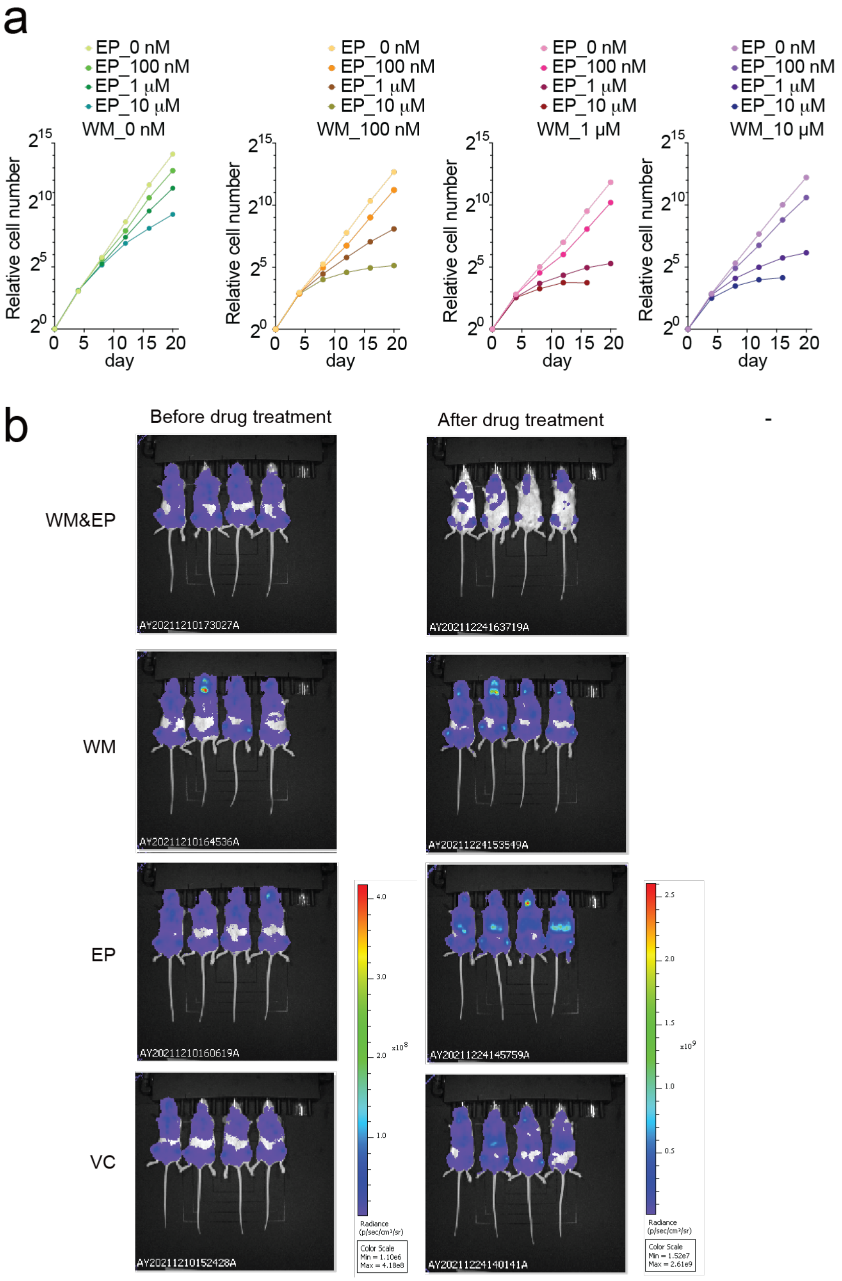

Supplementary Fig. 9 Combinatorial effects of the inhibitors for MOZ/MORF KATs and DOT1L KMT ex vivo and in vivo mentioned in Fig. 8. a, Effects on the proliferation of KP-Mo-TS cells, a leukemia cell line harboring CALM-AF10 fusion, by dual inhibition of MOZ/MORF KATs and DOT1L KMT. Proliferation in the presence of WM1119 and EPZ-5676 at the indicated concentrations was monitored for 21 days. **b**, Leukemia burden before and after the combination therapy with WM1119 and EPZ-5676. P31/FUJ human leukemia cells expressing LUC2Red were transplanted into sublethally irradiated SCID mice. Subsequently, the mice were administered with 35 mg/kg of WM1119 and 20 mg/kg of EPZ-5676 three times a day (i.p.) for two weeks. Representative images of luciferase-mediated bioluminescence reflecting leukemia cell abundance are shown with scale bars.

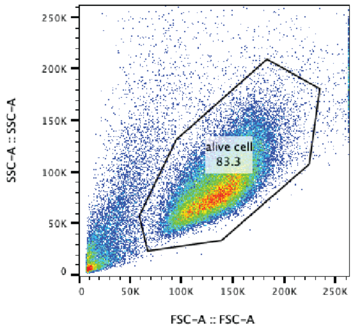

Supplementary Fig. 10 Gating strategy for alive cells, mentioned in FACS analysis of Methods

Alive cells were gated by the SSC/FSC profiles for the immunophenotype analysis.

Supplementary Table 1 sgRNA sequences for mouse genes

sgRNA	Sequence
Dot1l#2	AGTGGTTATATACCTGCTGC
Dot1l#3	ATGAGATTATTGAGACTATC
Enl#1	GAGCAGCTTGCAGCGAAACT
Enl#2	GCCCGACTCCTCCACTTTGT
Moz#1	CTCAATGCACTGCCACCGTA
Moz#2	TGCAGCTCCTGTCGTGACCA
Moz#9	GCTGTACACGGAGTGGATTT
Moz#11	GTACACGGAGTGGATTTTGG
Moz#12	GCTGCTCTAGCACAGTCCTG
Moz#13	GATGTATCTGCTGCCTGTGG
Moz#14	GAGGGCTTGGCAGAGACTGG
Morf#e11.1	CTTGTTAGCCAAGCTCTTCC
Morf#e12.1	AGGACACATTATACTTCTGC
Mor #1	ATTGTCTGGGTCCTTATATG
Morf#2	AAGCGTGCTGTGAACAATGG
Morf#3	TTACACAGAGTGGATTCTTG
Morf #4	GCAGAAGCAGCGGCCCTCAG
Morf #5	AGTGGATTCTTGAAGCAGTG
Hbo1#6	AGCCGCCGGCAATGCCGCGA
Hbo1#8	CCTCTCAGCTGACGAATGCA
Rpa3	GCTGGCGTTGACGCGCGCTT
Ren	GGATGATAACTGGTCCGCAG
Brpf1#1	TGTCTTGCGGACGCTAAAGG
Brpf1#2	CCAACCGCCTGACCATCCAG
Brpf1#3	CTGAAACGACAATCACGGAA
Brpf1#5	CATCCTCCTCCGAAAAACCT
Brpf1#6	CGAGCCGGTCCCTCTGTCTG

Supplementary Table 2 sgRNA sequences for human genes

sgRNA	Sequence
DOT1L#2	GACCGACGACGACCTGTTTG
DOT1L#3	CGTAGTTGTTGAGCTTCTCG
ENL#2	CTTGTACCGGAACTCCGTGG
ENL#6	GACTGGATGGTGTTTGTCCG
MOZ#2	GCAGAAACAGCGTCCTTCAG
MOZ#4	TTAGGAAGTGCTATTCGCCC
MORF#1	GTTGTCTGGGTCCTTATAGG
MORF#2	ATGCGGTCAGTACTTCCCAT
MORF#3	GGTTGTCTGGGTCCTTATAG
MORF#5	ATAGGAGGCAAGGCCTTTGT
HBO1#2	GCCGCTATGAGCTTGATACC
HBO1#4	GATCTGAGAGTGGACGTTCT
RPA3#6	GATGAATTGAGCTAGCATGC

Supplementary Table 3 sgRNA sequences used to generate knockout clones for human genes

sgRNA	Sequence
dDOT1L-Nick-F	TCTCGGGGTCGGTCACCGAG
dDOT1L-Nick-R	CTTCTCCCCCGAGGTGTACG
dMOZ-Nick-F	GCTATTCGCCCAGGATTATC
dMOZ-Nick-R	CTTCCTAAGCCTCGGAACCA

Supplementary Table 4 Custom qPCR probe/primer sequences

Target name	Forward	Reverse	Reporter
CD4- pre-TSS	TGTCCGAGCAAGGGATGATATTG	CCAAGTCACTCTGCACTACCA	ACTGCCACCATGCCAAT
CD4- post-TSS	GGACTCACTGTCCCTCCTGAA	CTTCAAGGCCATGAGGTCTCA	TCAGCCTTTCCGCCCTC
HOXA5 pre-TSS	ACACGAAAGAAAAATCGACTACGGTTA	AGGCGTCTATTAAAGATAGCTTTTGTGT	AAGGCGAGGTCAAATTCCAT
HOXA5 post-TSS	TGACCCGAAGGCCACTTG	GGCACAGCCCTCCCA	TTGCTTCCTGGGTTTCT
HOXA9 pre-TSS	TGGCTGCTTTTTTATGGCTTCAATT	CCGCGTGCGAGTGC	CCCCTCACATAAAATT
HOXA9 post-TSS	AGTGGCGGCGTAAATCCT	TGATCACGTCTGTGGCTTATTTGAA	CCCGCAGCCTCATC
MYC pre-TSS	GCGGTATCTGCTGCTTTGG	GCATTATGTATGCACAGCTATCTGGAT	CTGGGTGGAAGGTATCC
MYC post-TSS	GGGTAGGCGCAGGCA	GGTTTTTCCAAGTCAACGATTCCA	ATGTGTCCGATTCTCC
MYB pre-TSS	GGGCCCGGGTGTGT	GCCGCCACCGTCGTATT	TCACATGTCCTTCACTCTC
MYB post-TSS	CCCCCAGGCGAAAGGT	CTGAAGGAGGGTGCGAGAAG	CAGCCGCATCCACG
MEIS1 pre-TSS	CGGCGTTGATTCCCAATTTATTTCA	CACACAAACGCAGGCAGTAG	CCGCCAGCTTTATTTT
MEIS1 post-TSS	TCTCAGCGCCTCCAAATCTTG	TTTGTGTGTGTGAAATTTAGCTATTTAGGTTTT	CCAGGCAGTTATTTTC

Supplementary Table 5 Accession numbers of the NGS data

Sample name	DRA accession number	Sample ID	GEA accession number
cKit+BM-0226-1-RNA	DRA013593	SAMD00446086	E-GEAD-486
cKit+BM-0226-2-RNA	DRA013593	SAMD00446087	E-GEAD-486
cKit+BM-0225-RNA	DRA013593	SAMD00446088	E-GEAD-486
CALM-AF10-Ics-0131-RNA	DRA013593	SAMD00446089	E-GEAD-486
CALM-AF10-Ics-0820-RNA	DRA013593	SAMD00446090	E-GEAD-486
CALM-AF10-Ics-1223-RNA	DRA013593	SAMD00446091	E-GEAD-486
NES-ENL-Ics-0127-RNA	DRA013593	SAMD00446092	E-GEAD-486
NES-ENL-Ics-0203-RNA	DRA013593	SAMD00446093	E-GEAD-486
NES-ENL-Ics-0210-RNA	DRA013593	SAMD00446094	E-GEAD-486
DDX3X-AF10’-Ics-0106-RNA	DRA013593	SAMD00446095	E-GEAD-486
DDX3X-AF10’-Ics-0127-RNA	DRA013593	SAMD00446096	E-GEAD-486
DDX3X-AF10’-Ics-1230-RNA	DRA013593	SAMD00446097	E-GEAD-486
NUP98-AF10’-Ics-0106-RNA	DRA013593	SAMD00446098	E-GEAD-486
NUP98-AF10’-Ics-1028-RNA	DRA013593	SAMD00446099	E-GEAD-486
NUP98-AF10’-Ics-1230-RNA	DRA013593	SAMD00446100	E-GEAD-486
MLL-AF10-Ics-0210-1-RNA	DRA013593	SAMD00446101	E-GEAD-486
MLL-AF10-Ics-0210-2-RNA	DRA013593	SAMD00446102	E-GEAD-486
MLL-AF10-Ics-0210-3-RNA	DRA013593	SAMD00446103	E-GEAD-486

293T-fanChIP-INPUT(T0226_IN)	DRA004872	SAMD00055699	E-GEAD-320
293T-fanChIP-ENL rep1 (T0127_ENLx5)	DRA010819	SAMD00247200	E-GEAD-402
293T-fanChIP-MOZ (T1117_MYST3)	DRA008732	SAMD00180127	E-GEAD-322
293T-fanChIP-DOT1L (T0713_DOT1L)	DRA004872	SAMD00055697	E-GEAD-320
293T-fanChIP-AF4 (T1117_AF4)	DRA004872	SAMD00055708	E-GEAD-320
293T-fanChIP-RNAP2 non-P (T0329-parent)	DRA010819	SAMD00247201	E-GEAD-402
293T-fanChIP-RNAP2 Ser5-P(T0226_RNAP2 Ser5-P)	DRA004872	SAMD00055704	E-GEAD-320
293T-fanChIP-ENL rep 2 (T0226_ENL)	DRA013594	SAMD00446114	E-GEAD-487
293Tpa-fanChIP-IN(T0725_Tpa_IN)	DRA426544	SAMD00567561	E-GEAD-585
293TdMOZ-fanChIP-IN(T0715_TdMOZ_IN)	DRA426545	SAMD00567562	E-GEAD-585
293Tpa-fanChIP-ENL(T0725_Tpa_ENL)	DRA426546	SAMD00567563	E-GEAD-585
293TdMOZ-fanChIP-ENL(T0715_TdMOZ_ENL)	DRA426547	SAMD00567564	E-GEAD-585
293Tpa-fanChIP-DOT1L(T0809_Tpa_DOT1L)	DRA426548	SAMD00567565	E-GEAD-585
293TdMOZ-fanChIP-DOT1L(T00809_TdMOZ_DOT1L)	DRA426549	SAMD00567566	E-GEAD-585
P31-fanChIP-INPUT(P0224_IN)	DRA013594	SAMD00446115	E-GEAD-487
P31-fanChIP-ENL(P0217_ENL)	DRA013594	SAMD00446116	E-GEAD-487
P31-fanChIP-RNAP2 Ser5-P(P0217_RNAP2 Ser5-P)	DRA013594	SAMD00446117	E-GEAD-487

Supplementary Table 6 Key resources table

Reagent/Resource	Source	Identifier
**Antibodies**		
FLAG [WB 1000:1]	Sigma-Aldrich	Cat#F7425; RRID: AB_439687
FLAG (M2) [ChIP: 1 µg/ 400 µL]	Sigma-Aldrich	Cat#:F3165 RRID:AB_259529
Xpress [WB 1000:1]	Santa cruz Biotechnology	Cat#sc-499; RRID: AB_675764
HA (3F10) [WB 1000:1]	Roche	Cat#11867423001; RRID: AB_390918
DOT1L [ChIP: 1 µL/400 µL]	Bethyl Laboratories	Cat#A300-953A; RRID: AB_805775
DOT1L [WB 1000:1]	Cell Signaling Technology	Cat#77087; RRID: AB_2799889
MOZ [WB 1000:1, ChIP: 1 µL/400 µL]	Active motif	Cat#39868; Discontinued
MLL(N) [WB 1000:1]	Cell Signaling Technology	Cat# 14689 RRID: AB_2688009
MOZ [WB 1000:1, ChIP: 1 µL/400 µL]	Cell Signaling Technology	Cat#78462
CyclinT1 [WB 1000:1]	Bethyl Laboratories	Cat#A303-497A; RRID: AB_10952856
CyclinT1 [ChIP: 1 µg/400 µL]	Santa cruz Biotechnology	Cat#sc-8127 RRID: AB_2073892
ENL [WB 1000:1, ChIP:5 µg/400 µL]	Cell Signaling Technology	Cat#:14893S
RNAP2 Ser5-P [ChIP: 1 µg/400 µL]	Millipore	Cat#05-623; RRID: AB_309852
RNAP2 non-P [ChIP: 1 µg/400 µL]	Abcam	Cat#ab817; RRID: AB_306327

AF4 [ChIP: 1 µg/400 µL]	Santa cruz Biotechnology	Cat#sc-49350; RRID: AB_2226113
HBO1 [ChIP: 1 µg/400 µL]	Abcam	Cat#:70183 RRID:AB_1269226
ING4 [ChIP: 1 µg/400 µL]	Abcam	Cat#:108621 RRID:AB_10860023
GAPDH [WB 1000:1]	Cell Signaling Technology	Cat#1118
MORF [WB 1000:1]	Abcam	Cat#ab246879
Histone H3 [WB 5000:1]	Cell Signaling Technology	Cat#4499; RRID: AB_10544537
Histone H3K23ac [WB 5000:1]	Millipore (Upstate)	Cat#07-355; RRID: AB_310546
Histone H3K79me2 [WB 1000:1]	Cell Signaling Technology	Cat#5427; RRID: AB_10693787
Histone H3K4me2 [WB 1000:1]	Abcam	Cat#ab7766 RRID: AB_2560996
Histone H3K14ac [WB 1000:1]	Abcam	Cat#ab52946 RRID: AB_880442
PE-conjugated Mouse/Human CD11b [FACS:100:1]	Thermo Fisher Scientific	Cat#12-0112-82; RRID: AB_2734869
HRP-conjugated anti-rabbit IgG(H+L) [WB 5000:1]	Bethyl Laboratories	Cat#A120-201P
HRP-conjugated anti-mouse IgG(H+L) [WB 5000:1]	Bethyl Laboratories	Cat#A90-516P
HRP-conjugated anti-rat IgG(H+L) [WB 5000:1]	Bethyl Laboratories	Cat#A110-305P
**Bacterial and virus strains**		
**Chemicals, peptides, and recombinant proteins**		
WM1119	Enamine	Cat#:EN300-1719156
EPZ-5676	MCE	Cat# HY-15593
MI-2-2	Calbiochem	Cat#444825
MNase	Sigma-Aldrich	Cat#N3755-200U
Protease inhibitor cocktail	Roche	Cat#11873580001
Protein-G Magnetic beads	Thermo Fisher Scientific	Cat#1004D
mGM-CSF	Peprotech	Cat#500-P65
mIL3	Peprotech	Cat#213-13
mSCF	Peprotech	Cat#250-03
D-luciferin (VivoGlo Luciferin, *in vivo* grade,)	Promega	Cat#P1041
**Critical commercial assays**		
RNeasy Mini Kit	Qiagen	Cat#74106
SuperScript™ III First-Strand cDNA Synthesis System	Thermo Fisher Scientific	Cat#18080051
SureSelect Strand Specific RNA Library Prep Kit	Agilent Technologies	Cat#G9691A
TruSeq ChIP Sample Prep Kit SetB	Illumina	Cat#IP-202-1024
FLAG M2 antibody-conjugated beads	Sigma-Aldrich	Cat#M8823; RRID: AB_2637089
c-Kit magnetic beads	Miltenyi Biotec	Cat#130-091-224
ECL Prime Western Blotting Detection Reagent	GE Healthcare	Cat# 12316992
Lipofectamine 2000 transfection reagent	Thermo Fisher	Cat#11668027
MycoAlert Mycoplasma Detection Kit	Lonza	Cat#LT07-218
MethoCult™ H4435 Enriched	STEMCELL Technologies	Cat#: 04435
Stem Cell Culture Medium	Cell Applications, INC.	Cat#: 621-50
**Deposited data**		

Accession numbers of the NGS data (see Supplementary Table 5)		https://www.ddbj.nig.ac.jp/dra/index.html https://ddbj.nig.ac.jp/public/ddbj_database/gea/experiment/E-GEAD-000/
**Experimental models: Cell lines**		
Human: HB1119	Laboratory of Michael Cleary (ref. [2])	RRID: CVCL_8227
Human: HEK293T	Laboratory of Michael Cleary (ref. [3])	Authenticated by JRCB cell bank in 2019
Human: HEK293T	ATCC	Cat# CRL-3216, RRID:CVCL_0063
Human: HEK293T dMOZ#10	This paper	N/A
Human: HEK293T dMOZ#29	This paper	N/A
Human: HEK293T dDOT1L#14	This paper	N/A
Human: HEK293T dDOT1L#29	This paper	N/A
Human: PLAT-E	Laboratory of Toshio Kitamura (ref. [4])	N/A
Human: P31/FUJ	JCRB	Cat# JCRB0091, RRID:CVCL_1632
Human: MV4-11	ATCC	CRL-9591 RRID: CVCL_0064
Human: MonoMac-6	DSMZ	Cat# ACC-124, RRID:CVCL_1426
Human: OCI-AML3	DSMZ	Cat# ACC-582, RRID:CVCL_1844
Human: K562	Laboratory of Michael Cleary (ref. [2])	Authenticated by JRCB cell bank in 2021
Human: KP-Mo-TS	Laboratory of Issay Kitabayashi (ref.[5])	N/A
Human: CD34+ cells	Cell Applications INC.	Cat# 490L-01f
**Experimental models: Organisms/strains**		
Mouse: C57BL/6JJcl	CLEA Japan, Inc.	C57BL/6JJcl
Mouse: SCID (C.B-17/Icr-*scid/scid*Jcl)	CLEA Japan, Inc.	C.B-17/Icr-*scid/scid*Jcl)
**Oligonucleotides**		
Taqman probe for murine *Gapdh*	Life Technologies	Mm99999915_g1
Taqman probe for murine *Hoxa9*	Life Technologies	Mm00439364_m1
Taqman probe for human *GAPDH*	Life Technologies	Hs02786624_g1
Taqman probe for human *ACTB*	Life Technologies	Hs03023943_g1
Taqman probe for human *HOXA5*	Life Technologies	Hs00430330_m1
Taqman probe for human *HOXA9*	Life Technologies	Hs00365956_m1
Taqman probe for human *MYC*	Life Technologies	Hs01570247_m1
Taqman probe for human *MYB*	Life Technologies	Hs00920556_m1
Taqman probe for human *MEIS1*	Life Technologies	Hs00180020_m1
Taqman probe for human *ITGAM*	Life Technologies	Hs00167304_m1
sgRNA sequences used to generate knockout clones (see Supplementary Table 3)		
sgRNA sequences for mouse genes (see Supplementary Table 1)		
sgRNA sequences for human genes (see Supplementary Table 2)		
Custom qPCR probe/primer sequences (see Supplementary Table 4)		

**Recombinant DNA**		
pCMV5	Laboratory of Michael Cleary	N/A
pKLV2-EF1a-Cas9Bsd-W	ref. [6]	Addgene Plasmid #68343
pLKO-puro-FR-LUC	ref. [7]	N/A
pLKO5.sgRNA.EFS.GFP	ref. [8]	Addgene Plasmid #57822
pMD2.G	ref. [9]	Addgene Plasmid #12259
pMDLg/pRRE	ref. [9]	Addgene Plasmid #12251
pMSCV	Clontech	634401
pRSV-rev	ref. [9]	Addgene Plasmid #12253
pCDH-MSCV-MCS-EF1-Puro	System Biosciences	Cat#CD710B-1
**Software and algorithms**		
BWA	ref. [10]	http://bio-bwa.sourceforge.net
Cutadapt	ref. [11]	https://cutadapt.readthedocs.io/en/stable/index.html
FlowJo	BD Biosciences	N/A
GraphPad Prism 8	GraphPad Software Inc.	N/A
Integrative Genomics Viewer	ref. [12]	https://software.broadinstitute.org/software/igv/
ngsplot	ref. [1]	https://github.com/shenlab-sinai/ngsplot
CCTop	ref. [13]	https://cctop.cos.uni-heidelberg.de:8043
STAR	ref. [14]	https://github.com/alexdobin/STAR
featureCounts	ref. [15]	https://bioconductor.org/packages/release/bioc/html/Rsubread.html
TCC R package	refs. [16,17]	https://bioconductor.org/packages/release/bioc/html/TCC.html
ComplexHeatmap R package	ref. [18]	https://bioconductor.org/packages/release/bioc/html/ComplexHeatmap.html
ImageJ	NIH	https://imagej.nih.gov/ij/
Metascape	ref. [19]	https://metascape.org/gp/index.html#/main/step1
GSEA	ref.[20]	https://www.gsea-msigdb.org/gsea/index.jsp
**Other**		

Uncropped scans of western blotting data in this supplementary information

Supplementary Fig.1

a

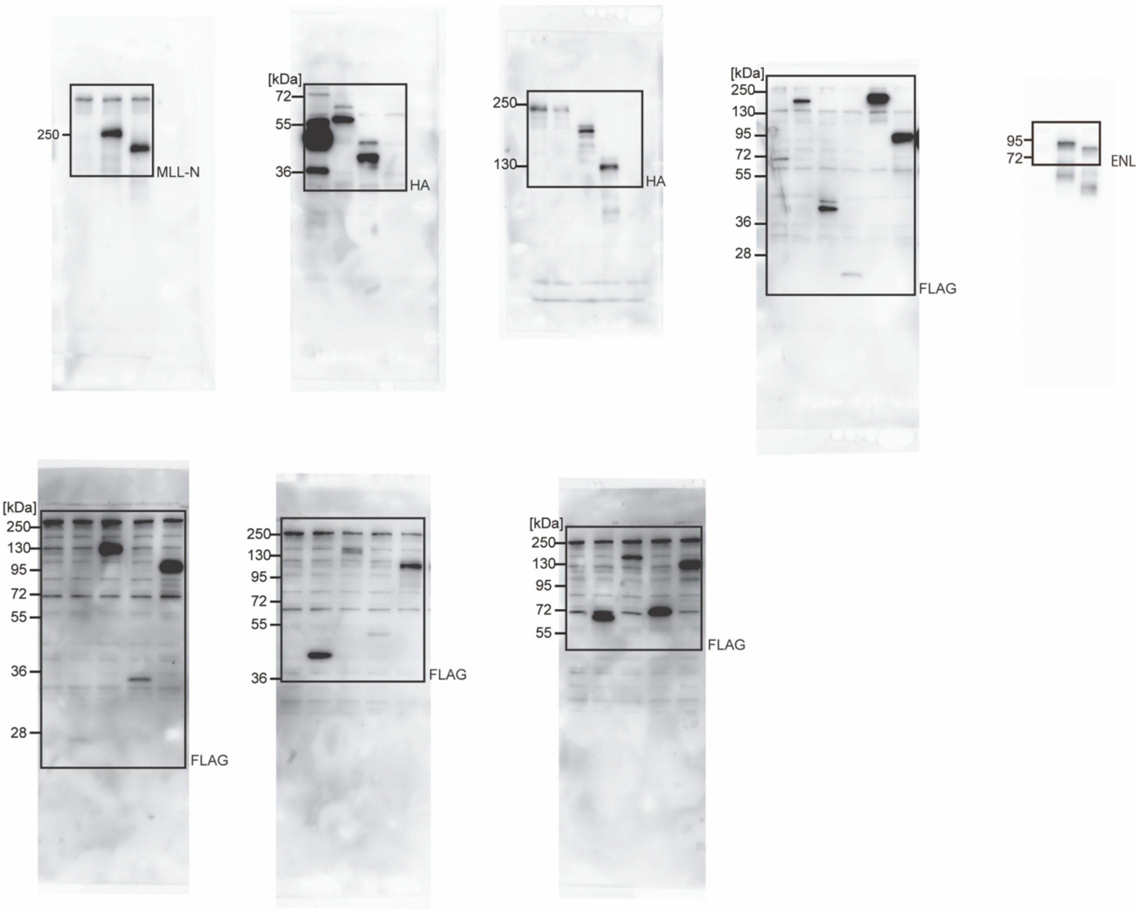

Supplementary Fig.4

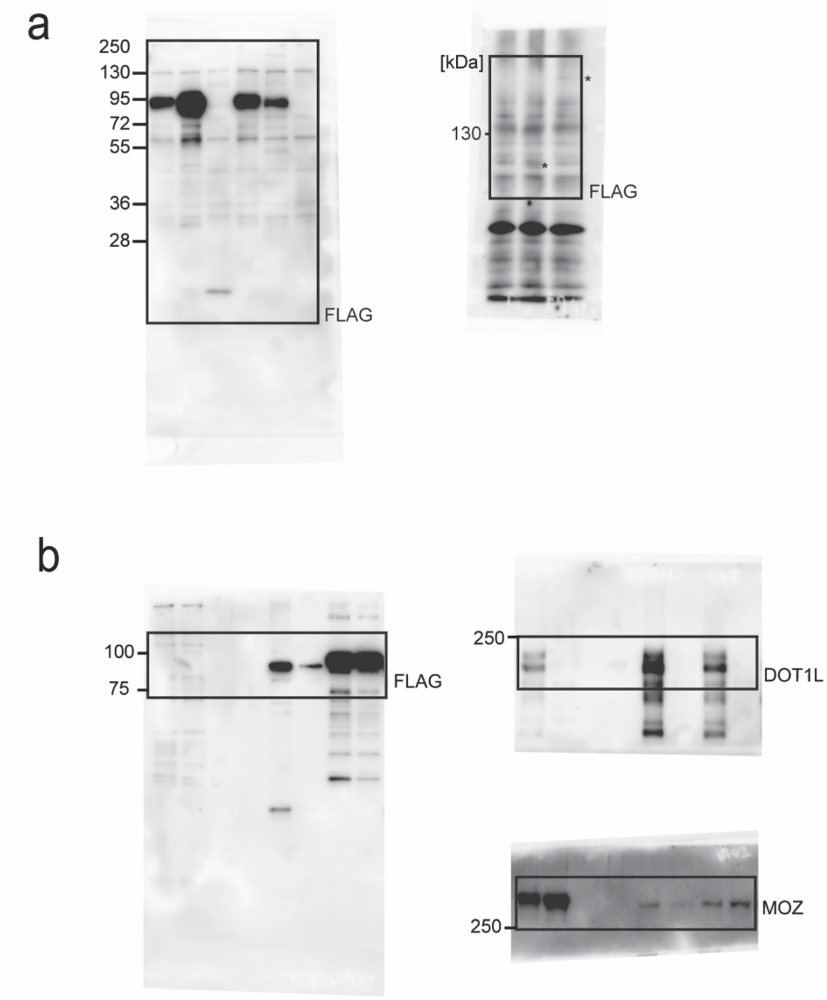

Supplementary Fig.6

c

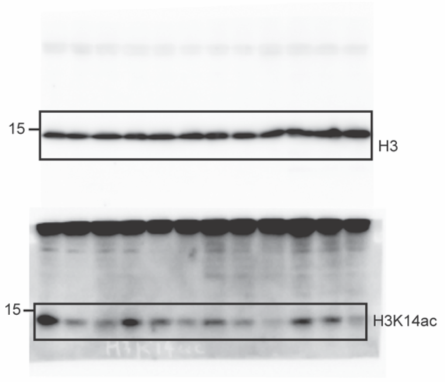

d

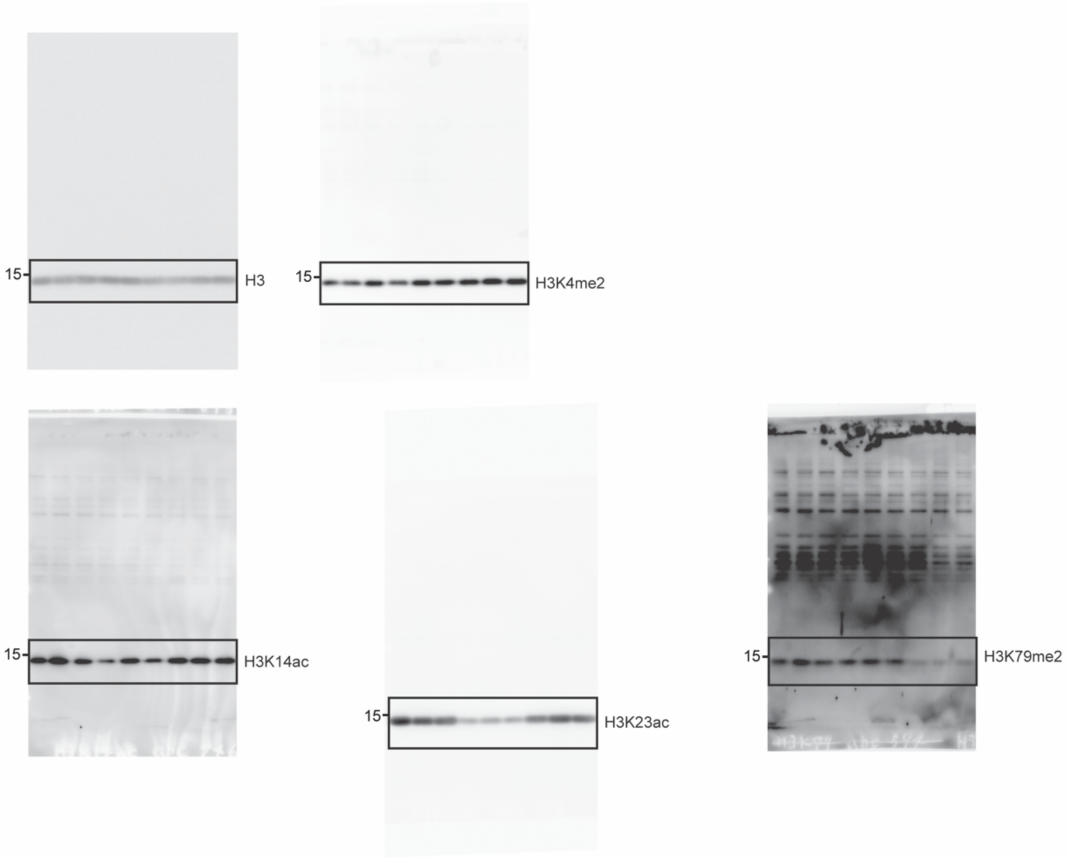

Supplementary Fig.8

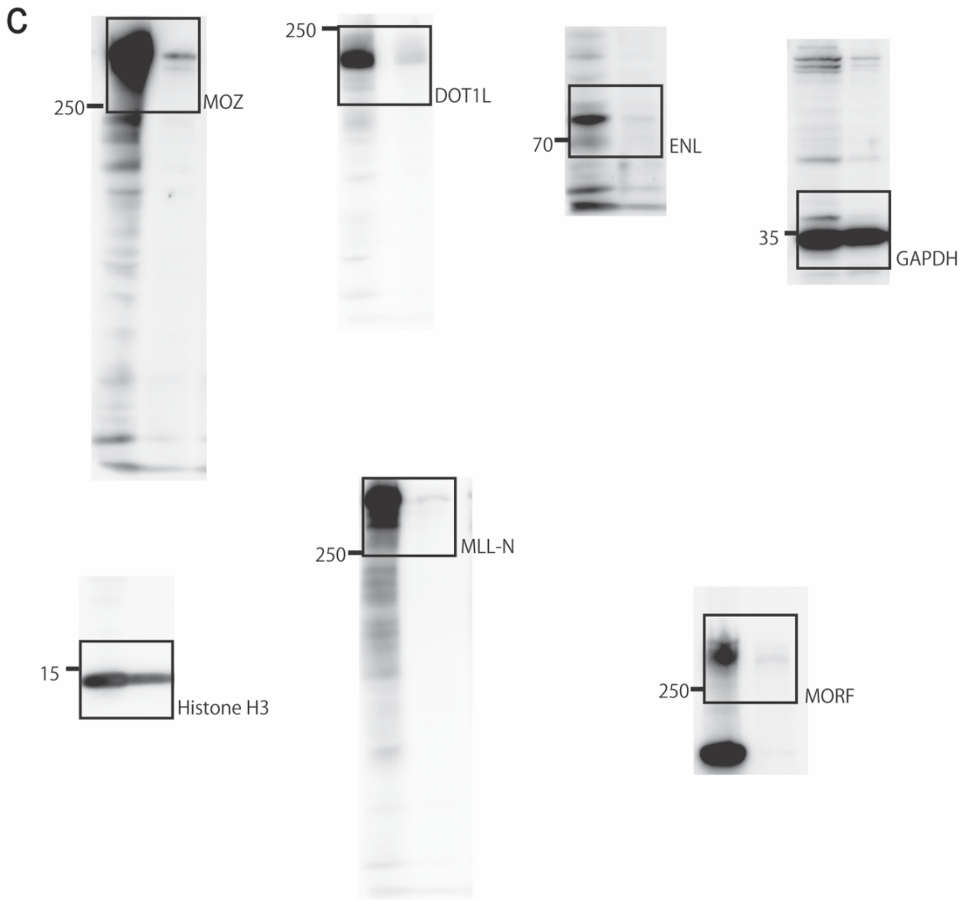

References

1. Shen, L., Shao, N., Liu, X. & Nestler, E. ngs.plot: Quick mining and visualization of next-generation sequencing data by integrating genomic databases. *BMC Genomics* **15**, 284 (2014).
2. Tkachuk, D.C., Kohler, S. & Cleary, M.L. Involvement of a homolog of Drosophila trithorax by 11q23 chromosomal translocations in acute leukemias. *Cell* **71**, 691-700 (1992).
3. Yokoyama, A. *et al.* Leukemia proto-oncoprotein MLL forms a SET1-like histone methyltransferase complex with menin to regulate Hox gene expression. *Mol Cell Biol* **24**, 5639-49 (2004).
4. Morita, S., Kojima, T. & Kitamura, T. Plat-E: an efficient and stable system for transient packaging of retroviruses. *Gene Ther* **7**, 1063-6 (2000).
5. Narita, M. *et al.* Consistent detection of CALM-AF10 chimaeric transcripts in haematological malignancies with t(10;11)(p13;q14) and identification of novel transcripts. *Br J Haematol* **105**, 928-37 (1999).
6. Tzelepis, K. *et al.* A CRISPR Dropout Screen Identifies Genetic Vulnerabilities and Therapeutic Targets in Acute Myeloid Leukemia. *Cell Rep* **17**, 1193-1205 (2016).
7. Okuda, H., Kanai, A., Ito, S., Matsui, H. & Yokoyama, A. AF4 uses the SL1 components of RNAP1 machinery to initiate MLL fusion- and AEP-dependent transcription. *Nat Commun* **6**, 8869 (2015).
8. Heckl, D. *et al.* Generation of mouse models of myeloid malignancy with combinatorial genetic lesions using CRISPR-Cas9 genome editing. *Nat Biotechnol* **32**, 941-6 (2014).
9. Dull, T. *et al.* A third-generation lentivirus vector with a conditional packaging system. *J Virol* **72**, 8463-71 (1998).
10. Li, H. & Durbin, R. Fast and accurate short read alignment with Burrows-Wheeler transform. *Bioinformatics* **25**, 1754-60 (2009).
11. Martin, M. Cutadapt Removes Adapter Sequences From High-Throughput Sequencing Reads. *EMBnet Journal* **17**, 10-12 (2011).
12. Thorvaldsdottir, H., Robinson, J.T. & Mesirov, J.P. Integrative Genomics Viewer (IGV): high-performance genomics data visualization and exploration. *Brief Bioinform* **14**, 178-92 (2013).

13. Stemmer, M., Thumberger, T., Del Sol Keyer, M., Wittbrodt, J. & Mateo, J.L. CCTop: An Intuitive, Flexible and Reliable CRISPR/Cas9 Target Prediction Tool. *PLoS One* **10**, e0124633 (2015).
14. Dobin, A. *et al.* STAR: ultrafast universal RNA-seq aligner. *Bioinformatics* **29**, 15-21 (2013).
15. Liao, Y., Smyth, G.K. & Shi, W. featureCounts: an efficient general purpose program for assigning sequence reads to genomic features. *Bioinformatics* **30**, 923-30 (2014).
16. Robinson, M.D., McCarthy, D.J. & Smyth, G.K. edgeR: a Bioconductor package for differential expression analysis of digital gene expression data. *Bioinformatics* **26**, 139-40 (2010).
17. Sun, J., Nishiyama, T., Shimizu, K. & Kadota, K. TCC: an R package for comparing tag count data with robust normalization strategies. *BMC Bioinformatics* **14**, 219 (2013).
18. Gu, Z., Eils, R. & Schlesner, M. Complex heatmaps reveal patterns and correlations in multidimensional genomic data. *Bioinformatics* **32**, 2847-9 (2016).
19. Zhou, Y. *et al.* Metascape provides a biologist-oriented resource for the analysis of systems-level datasets. *Nat Commun* **10**, 1523 (2019).
20. Subramanian, A. *et al.* Gene set enrichment analysis: a knowledge-based approach for interpreting genome-wide expression profiles. *Proc Natl Acad Sci U S A* **102**, 15545-50 (2005).
