# Supplementary material for: MOZ/ENL complex is a recruiting factor of leukemic AF10 fusion proteins
Source: Nat Commun. 2023 Apr 8;14:1979. doi: 10.1038/s41467-023-37712-5 (PMC10082848; doi:10.1038/s41467-023-37712-5)
Supplement: Supplementary file 3 — Reporting Summary [file 41467_2023_37712_MOESM3_ESM.pdf]

nature portfolio

Corresponding author(s): Akihiko Yokoyama
Last updated by author(s): Mar 10, 2023

Reporting Summary

Nature Portfolio wishes to improve the reproducibility of the work that we publish. This form provides structure for consistency and transparency in reporting. For further information on Nature Portfolio policies, see our Editorial Policies and the Editorial Policy Checklist.

Statistics

For all statistical analyses, confirm that the following items are present in the figure legend, table legend, main text, or Methods section.

n/a	Confirmed	
☐	☒	The exact sample size (*n*) for each experimental group/condition, given as a discrete number and unit of measurement
☐	☒	A statement on whether measurements were taken from distinct samples or whether the same sample was measured repeatedly
☐	☒	The statistical test(s) used AND whether they are one- or two-sided<br>*Only common tests should be described solely by name; describe more complex techniques in the Methods section.*
☐	☒	A description of all covariates tested
☒	☐	A description of any assumptions or corrections, such as tests of normality and adjustment for multiple comparisons
☐	☒	A full description of the statistical parameters including central tendency (e.g. means) or other basic estimates (e.g. regression coefficient) AND variation (e.g. standard deviation) or associated estimates of uncertainty (e.g. confidence intervals)
☐	☒	For null hypothesis testing, the test statistic (e.g. *F*, *t*, *r*) with confidence intervals, effect sizes, degrees of freedom and *P* value noted<br>*Give P values as exact values whenever suitable.*
☒	☐	For Bayesian analysis, information on the choice of priors and Markov chain Monte Carlo settings
☒	☐	For hierarchical and complex designs, identification of the appropriate level for tests and full reporting of outcomes
☐	☒	Estimates of effect sizes (e.g. Cohen's *d*, Pearson's *r*), indicating how they were calculated

Our web collection on statistics for biologists contains articles on many of the points above.

Software and code

Policy information about availability of computer code

Data collection	RT-qPCR data were collected with StepOnePlus or QuantStudio 3 Real time PCR systems (Thermo Fisher). FACS data were collected with FACSmelody Cell Sorter with FACSChorus software ver.1.3 (BD Biosciences). Western blotting data were collected with FUSION FX imaging system (VILBER).
Data analysis	All statistical data were analyzed with Prism 8 software (GraphPad software) or MIcrosoft Excel for Mac (ver16. 67). All FACS data were analyzed with FlowJo software (v.10). Western blotting data were analyzed using Image J software (ver 1.53a)

For manuscripts utilizing custom algorithms or software that are central to the research but not yet described in published literature, software must be made available to editors and reviewers. We strongly encourage code deposition in a community repository (e.g. GitHub). See the Nature Portfolio guidelines for submitting code & software for further information.

Data

Policy information about availability of data

All manuscripts must include a data availability statement. This statement should provide the following information, where applicable:

- Accession codes, unique identifiers, or web links for publicly available datasets
- A description of any restrictions on data availability
- For clinical datasets or third party data, please ensure that the statement adheres to our policy

All data supporting the findings of this study are available within the article, in the supplementary information, and in the source data. ChIP-seq and RNA-seq data have been deposited at the DDBJ (DNA Data Bank of Japan) Sequence Read Archive as fastq files [https://ddbj.nig.ac.jp/public/ddbj_database/dra/fastq/] and as

WIG files [https://ddbj.nig.ac.jp/public/ddbj_database/gea/experiment/E-GEAD-000/] under the accession numbers and sample IDs listed in Supplementary Table 5. Further information and requests for resources and reagents should be directed to and will be fulfilled by Akihiko Yokoyama (ayokoyam@ncc-tmc.jp). Source data are provided with this paper.

Sample name, DRA accession number, and Sample ID GEA accession number
cKit+BM-0226-1-RNA DRA013593 SAMD00446086 E-GEAD-486
cKit+BM-0226-2-RNA DRA013593 SAMD00446087 E-GEAD-486
cKit+BM-0225-RNA DRA013593 SAMD00446088 E-GEAD-486
CALM-AF10-lcs-0131-RNA DRA013593 SAMD00446089 E-GEAD-486
CALM-AF10-lcs-0820-RNA DRA013593 SAMD00446090 E-GEAD-486
CALM-AF10-lcs-1223-RNA DRA013593 SAMD00446091 E-GEAD-486
NES-ENL-lcs-0127-RNA DRA013593 SAMD00446092 E-GEAD-486
NES-ENL-lcs-0203-RNA DRA013593 SAMD00446093 E-GEAD-486
NES-ENL-lcs-0210-RNA DRA013593 SAMD00446094 E-GEAD-486
DDX3X-AF10'-lcs-0106-RNA DRA013593 SAMD00446095 E-GEAD-486
DDX3X-AF10'-lcs-0127-RNA DRA013593 SAMD00446096 E-GEAD-486
DDX3X-AF10'-lcs-1230-RNA DRA013593 SAMD00446097 E-GEAD-486
NUP98-AF10'-lcs-0106-RNA DRA013593 SAMD00446098 E-GEAD-486
NUP98-AF10'-lcs-1028-RNA DRA013593 SAMD00446099 E-GEAD-486
NUP98-AF10'-lcs-1230-RNA DRA013593 SAMD00446100 E-GEAD-486
MLL-AF10-lcs-0210-1-RNA DRA013593 SAMD00446101 E-GEAD-486
MLL-AF10-lcs-0210-2-RNA DRA013593 SAMD00446102 E-GEAD-486
MLL-AF10-lcs-0210-3-RNA DRA013593 SAMD00446103 E-GEAD-486
293T-fanChIP-INPUT(T0226_IN) DRA004872 SAMD00055699 E-GEAD-320
293T-fanChIP-ENL rep1 (T0127_ENLx5) DRA010819 SAMD00247200 E-GEAD-402
293T-fanChIP-MOZ (T1117_MYST3) DRA008732 SAMD00180127 E-GEAD-322
293T-fanChIP-DOT1L (T0713_DOT1L) DRA004872 SAMD00055697 E-GEAD-320
293T-fanChIP-AF4 (T1117_AF4) DRA004872 SAMD00055708 E-GEAD-320
293T-fanChIP-RNAP2 non-P (T0329-parent) DRA010819 SAMD00247201 E-GEAD-402
293T-fanChIP-RNAP2 Ser5-P(T0226_RNAP2 Ser5-P) DRA004872 SAMD00055704 E-GEAD-320
293T-fanChIP-ENL rep 2 (T0226_ENL) DRA013594 SAMD00446114 E-GEAD-487
P31-fanChIP-INPUT(P0224_IN) DRA013594 SAMD00446115 E-GEAD-487
P31-fanChIP-ENL(P0217_ENL) DRA013594 SAMD00446116 E-GEAD-487
P31-fanChIP-RNAP2 Ser5-P(P0217_RNAP2 Ser5-P) DRA013594 SAMD00446117 E-GEAD-487
293Tpa-fanChIP-IN(T0725_Tpa_IN) DRA426544 SAMD00567561 E-GEAD-585
293TdMOZ-fanChIP-IN(T0715_TdMOZ_IN) DRA426545 SAMD00567562 E-GEAD-585
293Tpa-fanChIP-ENL(T0725_Tpa_ENL) DRA426546 SAMD00567563 E-GEAD-585
293TdMOZ-fanChIP-ENL(T0715_TdMOZ_ENL) DRA426547 SAMD00567564 E-GEAD-585
293Tpa-fanChIP-DOT1L(T0809_Tpa_DOT1L) DRA426548 SAMD00567565 E-GEAD-585
293TdMOZ-fanChIP-DOT1L(T00809_TdMOZ_DOT1L) DRA426549 SAMD00567566 E-GEAD-585

Human research participants

Policy information about studies involving human research participants and Sex and Gender in Research.

Reporting on sex and gender	N/A
Population characteristics	N/A
Recruitment	N/A
Ethics oversight	N/A

Note that full information on the approval of the study protocol must also be provided in the manuscript.

Field-specific reporting

Please select the one below that is the best fit for your research. If you are not sure, read the appropriate sections before making your selection.

[x] Life sciences [ ] Behavioural & social sciences [ ] Ecological, evolutionary & environmental sciences

For a reference copy of the document with all sections, see nature.com/documents/nr-reporting-summary-flat.pdf

Life sciences study design

All studies must disclose on these points even when the disclosure is negative.

Sample size	The chosen sample size are based on the numbers used for previous publications.<br>Okuda H, Miyamoto M, Takahashi S, Kawamura T Ichikawa J, Harada I, Tamura T and Yokoyama A. RNA-Binding Proteins of KHDRBS and IGF2BP families control the Oncogenic Activity of MLL-AF4 Nature Communications (2022) 13:6688<br>Miyamoto R, Okuda H, Kanai A, Takahashi S, Kawamura T, Matsui H, Kitamura T, Kitabayashi I, Inaba T, Yokoyama A Activation of CpG-rich promoters mediated by MLL drives MOZ-rearranged leukemia Cell Reports 32:13;108200 (2020)
Data exclusions	No data were excluded from the analyses.
Replication	All of the IP -western, ChIP-qPCR, RT-qPCR, and gRNA competition assay experiments were performed at least twice and confirmed their reproducibility. Myeloid progenitor transformation assay, and drug sensitivity analysis were performed at least three times (>3 biological replicates). RNA-seq analysis was performed for three biological replicates for each sample type. ChIP-seq analysis was performed once for most of the samples, and its reproducibility was confirmed by ChIP-qPCR analysis on selected targets. ChIP-seq analysis for ENL in HEK293T was performed twice for reproducibility. The other experiments were performed at least twice.
Randomization	Randomization was not applied to the in vivo drug sensitivity tests, as the mice carrying leukemia cells were evenly distributed to each experimental group based on the luminescent signals before the drug treatment.
Blinding	Investigators were not blinded to the sample identities during data collection as the data were independently collected by technicians

Behavioural & social sciences study design

All studies must disclose on these points even when the disclosure is negative.

Study description	*Briefly describe the study type including whether data are quantitative, qualitative, or mixed-methods (e.g. qualitative cross-sectional, quantitative experimental, mixed-methods case study).*
Research sample	*State the research sample (e.g. Harvard university undergraduates, villagers in rural India) and provide relevant demographic information (e.g. age, sex) and indicate whether the sample is representative. Provide a rationale for the study sample chosen. For studies involving existing datasets, please describe the dataset and source.*
Sampling strategy	*Describe the sampling procedure (e.g. random, snowball, stratified, convenience). Describe the statistical methods that were used to predetermine sample size OR if no sample-size calculation was performed, describe how sample sizes were chosen and provide a rationale for why these sample sizes are sufficient. For qualitative data, please indicate whether data saturation was considered, and what criteria were used to decide that no further sampling was needed.*
Data collection	*Provide details about the data collection procedure, including the instruments or devices used to record the data (e.g. pen and paper, computer, eye tracker, video or audio equipment) whether anyone was present besides the participant(s) and the researcher, and whether the researcher was blind to experimental condition and/or the study hypothesis during data collection.*
Timing	*Indicate the start and stop dates of data collection. If there is a gap between collection periods, state the dates for each sample cohort.*
Data exclusions	*If no data were excluded from the analyses, state so OR if data were excluded, provide the exact number of exclusions and the rationale behind them, indicating whether exclusion criteria were pre-established.*
Non-participation	*State how many participants dropped out/declined participation and the reason(s) given OR provide response rate OR state that no participants dropped out/declined participation.*
Randomization	*If participants were not allocated into experimental groups, state so OR describe how participants were allocated to groups, and if allocation was not random, describe how covariates were controlled.*

Ecological, evolutionary & environmental sciences study design

All studies must disclose on these points even when the disclosure is negative.

Study description	*Briefly describe the study. For quantitative data include treatment factors and interactions, design structure (e.g. factorial, nested, hierarchical), nature and number of experimental units and replicates.*
Research sample	*Describe the research sample (e.g. a group of tagged Passer domesticus, all Stenocereus thurberi within Organ Pipe Cactus National Monument), and provide a rationale for the sample choice. When relevant, describe the organism taxa, source, sex, age range and any manipulations. State what population the sample is meant to represent when applicable. For studies involving existing datasets,*

	describe the data and its source.
Sampling strategy	*Note the sampling procedure. Describe the statistical methods that were used to predetermine sample size OR if no sample-size calculation was performed, describe how sample sizes were chosen and provide a rationale for why these sample sizes are sufficient.*
Data collection	*Describe the data collection procedure, including who recorded the data and how.*
Timing and spatial scale	*Indicate the start and stop dates of data collection, noting the frequency and periodicity of sampling and providing a rationale for these choices. If there is a gap between collection periods, state the dates for each sample cohort. Specify the spatial scale from which the data are taken*
Data exclusions	*If no data were excluded from the analyses, state so OR if data were excluded, describe the exclusions and the rationale behind them, indicating whether exclusion criteria were pre-established.*
Reproducibility	*Describe the measures taken to verify the reproducibility of experimental findings. For each experiment, note whether any attempts to repeat the experiment failed OR state that all attempts to repeat the experiment were successful.*
Randomization	*Describe how samples/organisms/participants were allocated into groups. If allocation was not random, describe how covariates were controlled. If this is not relevant to your study, explain why.*
Blinding	*Describe the extent of blinding used during data acquisition and analysis. If blinding was not possible, describe why OR explain why blinding was not relevant to your study.*

Did the study involve field work? ☐ Yes ☐ No

Field work, collection and transport

Field conditions	*Describe the study conditions for field work, providing relevant parameters (e.g. temperature, rainfall).*
Location	*State the location of the sampling or experiment, providing relevant parameters (e.g. latitude and longitude, elevation, water depth).*
Access & import/export	*Describe the efforts you have made to access habitats and to collect and import/export your samples in a responsible manner and in compliance with local, national and international laws, noting any permits that were obtained (give the name of the issuing authority, the date of issue, and any identifying information).*
Disturbance	*Describe any disturbance caused by the study and how it was minimized.*

Reporting for specific materials, systems and methods

We require information from authors about some types of materials, experimental systems and methods used in many studies. Here, indicate whether each material, system or method listed is relevant to your study. If you are not sure if a list item applies to your research, read the appropriate section before selecting a response.

Materials & experimental systems

n/a	Involved in the study	
☐	☒	Antibodies
☐	☒	Eukaryotic cell lines
☒	☐	Palaeontology and archaeology
☐	☒	Animals and other organisms
☒	☐	Clinical data
☒	☐	Dual use research of concern

Methods

n/a	Involved in the study	
☐	☒	ChIP-seq
☐	☒	Flow cytometry
☒	☐	MRI-based neuroimaging

Antibodies

Antibodies used	Antibodies<br>FLAG [WB 1000:1] Sigma-Aldrich Cat#F7425; RRID: AB_439687<br>FLAG (M2) [ChIP: 1 μg/ 400 μL] Sigma-Aldrich Cat#:F3165; RRID:AB_259529<br>Xpress [WB 1000:1] Santa cruz Biotechnology Cat#sc-499; RRID: AB_675764<br>HA (3F10) [WB 1000:1] Roche Cat#11867423001; RRID: AB_390918<br>DOT1L [ChIP: 1 μL/400 μL] Bethyl Laboratories Cat#A300-953A; RRID: AB_805775<br>DOT1L [WB 1000:1] Cell Signaling Technology Cat#77087; RRID: AB_2799889<br>MOZ [WB 1000:1, ChIP: 1 μL/400 μL] Active motif Cat#39868; Discontinued

MLL(N) [WB 1000:1] Cell Signaling Technology Cat# 14689; RRID: AB_2688009
MOZ [WB 1000:1, ChIP: 1 µL/400 µL] Cell Signaling Technology Cat#78462
CyclinT1 [WB 1000:1] Bethyl Laboratories Cat#A303-497A; RRID: AB_10952856
CyclinT1 [ChIP: 1 µg/400 µL] Santa cruz Biotechnology Cat#sc-8127; RRID: AB_2073892
ENL [WB 1000:1, ChIP:5 µg/400 µL] Cell Signaling Technology Cat#:14893S
RNAP2 Ser5-P [ChIP: 1 µg/400 µL] Millipore Cat#05-623; RRID: AB_309852
RNAP2 non-P [ChIP: 1 µg/400 µL] Abcam Cat#ab817; RRID: AB_306327
AF4 [ChIP: 1 µg/400 µL] Santa cruz Biotechnology Cat#sc-49350; RRID: AB_2226113
HBO1 [ChIP: 1 µg/400 µL] Abcam Cat#:70183; RRID:AB_1269226
ING4 [ChIP: 1 µg/400 µL] Abcam Cat#:108621; RRID:AB10860023
Histone H3 [WB 5000:1] Cell Signaling Technology Cat#4499;RRID: AB_10544537
Histone H3K23ac [WB 5000:1] Millipore (Upstate) Cat#07-355; RRID: AB_310546
Histone H3K79me2 [WB 1000:1] Cell Signaling Technology Cat#5427; RRID: AB_10693787
Histone H3K4me2 [WB 1000:1] Abcam Cat#ab7766; RRID: AB_2560996
Histone H3K14ac [WB 1000:1] Abcam Cat#ab52946; RRID: AB_880442
GAPDH [WB 1000:1] Cell Signaling Technology Cat#1118
MORF [WB 1000:1] Abcam Cat#ab246879
PE-conjugated Mouse/Human CD11b [FACS:100:1] Thermo Fisher Scientific Cat#12-0112-82; RRID: AB_2734869
HRP-conjugated anti-rabbit IgG(H+L) [WB 5000:1] Bethyl Laboratories Cat#A120-201P
HRP-conjugated anti-mouse IgG(H+L) [WB 5000:1] Bethyl Laboratories Cat#A90-516P
HRP-conjugated anti-rat IgG(H+L) [WB 5000:1] Bethyl Laboratories Cat#A110-305P

Validation

Antibodies were validated to be reactive to the target proteins of Human species by manufacturers and our previous publications.
Takahashi S, Kanai A, Okuda H, Miyamoto R, Komata Y, Kawamura T, Matsui H, Inaba T, Takaori-Kondo A, Yokoyama A. HBO1-MLL interaction promotes AF4/ENL/P-TEFb-mediated leukemogenesis. eLife 10:e65872 (2021)
Miyamoto R, Okuda H, Kanai A, Takahashi S, Kawamura T, Matsui H, Kitamura T, Kitabayashi I, Inaba T, Yokoyama A Activation of CpG-rich promoters mediated by MLL drives MOZ-rearranged leukemia Cell Reports 32:13;108200 (2020)
Okuda H, Stanojevic B, Kanai A, Kawamura T, Takahashi S, Matsui H, Takaori-Kondo A, Yokoyama A. Cooperative gene activation by AF4 and DOT1L drives MLL-rearranged leukemia. J. Clin. Invest. 127(5) 1918-1931(2017)

Eukaryotic cell lines

Policy information about cell lines and Sex and Gender in Research

Cell line source(s)

Experimental models: Cell lines
Human: HB1119 Laboratory of Michael Cleary RRID: CVCL_8227
Human: HEK293T Laboratory of Michael Cleary
Human: HEK293T ATCC Cat# CRL-3216, RRID:CVCL_0063
Human: HEK293T dMOZ#10 This paper
Human: HEK293T dMOZ#29 This paper
Human: HEK293T dDOT1L#14 This paper
Human: HEK293T dDOT1L#29 This paper
Human: PLAT-E Laboratory of Toshio Kitamura
Human: P31/FUJ JCRB Cat# JCRB0091, RRID:CVCL_1632
Human: MV4-11 ATCC CRL-9591RRID: CVCL_0064
Human: MonoMac-6 DSMZ Cat# ACC-124, RRID:CVCL_1426
Human: OCI-AML3 DSMZ Cat# ACC-582, RRID:CVCL_1844
Human: K562 Laboratory of Michael Cleary (The original source is unknown but authenticated by JRCB cell bank in 2021)
Human: KP-Mo-TS Laboratory of Issay Kitabayashi (The original source is Laboratory of Misao Ohki)
Human CD34+ cells, Cell Applications INC., Cat# 490L-01f

Authentication

HEK293T and K562 cells were authenticated by JRCB cell bank by STR profiling.
HB1119 cells were routinely checked for the expression of MLL-ENL protein that is specifically expressed from the rearranged allele.
Other cell lines were authenticated by the providers (ATCC, DSMZ and JCRB)

Mycoplasma contamination

All cell lines tested negative for mycoplasma contamination.

Commonly misidentified lines
(See ICLAC register)

No commonly misidentified lines were used.

Palaeontology and Archaeology

Specimen provenance

Provide provenance information for specimens and describe permits that were obtained for the work (including the name of the issuing authority, the date of issue, and any identifying information). Permits should encompass collection and, where applicable,

	export.
Specimen deposition	*Indicate where the specimens have been deposited to permit free access by other researchers.*
Dating methods	*If new dates are provided, describe how they were obtained (e.g. collection, storage, sample pretreatment and measurement), where they were obtained (i.e. lab name), the calibration program and the protocol for quality assurance OR state that no new dates are provided.*

☐ Tick this box to confirm that the raw and calibrated dates are available in the paper or in Supplementary Information.

Ethics oversight	*Identify the organization(s) that approved or provided guidance on the study protocol, OR state that no ethical approval or guidance was required and explain why not.*

Note that full information on the approval of the study protocol must also be provided in the manuscript.

Animals and other research organisms

Policy information about studies involving animals; ARRIVE guidelines recommended for reporting animal research, and Sex and Gender in Research

Laboratory animals	Female C57BL/6JJcl mice were obtained from CLEA Japan inc.. Five-week-old female C57BL/6JJcl mice were used for bone marrow extraction and seven to eight-week-old mice were used for leukemogenesis assay. Mice were allowed free access to food and water and were maintained at room temperature (about 25C) with constant humidity (about 50%) on a 12-hours light/dark cycle.
Wild animals	No wild animals were used.
Reporting on sex	Female mice were used.
Field-collected samples	No field-collected samples were used.
Ethics oversight	All the animal experimental protocols were approved by the Institutional Animal Care and Use Committee of the National Cancer Center (Tokyo, Japan) and performed by abiding by ethical regulations

Note that full information on the approval of the study protocol must also be provided in the manuscript.

Clinical data

Policy information about clinical studies

All manuscripts should comply with the ICMJEguidelines for publication of clinical research and a completedCONSORT checklist must be included with all submissions.

Clinical trial registration	*Provide the trial registration number from ClinicalTrials.gov or an equivalent agency.*
Study protocol	*Note where the full trial protocol can be accessed OR if not available, explain why.*
Data collection	*Describe the settings and locales of data collection, noting the time periods of recruitment and data collection.*
Outcomes	*Describe how you pre-defined primary and secondary outcome measures and how you assessed these measures.*

Dual use research of concern

Policy information about dual use research of concern

Hazards

Could the accidental, deliberate or reckless misuse of agents or technologies generated in the work, or the application of information presented in the manuscript, pose a threat to:

No	Yes	
☐	☐	Public health
☐	☐	National security
☐	☐	Crops and/or livestock
☐	☐	Ecosystems
☐	☐	Any other significant area

Experiments of concern

Does the work involve any of these experiments of concern:

No	Yes	
☐	☐	Demonstrate how to render a vaccine ineffective
☐	☐	Confer resistance to therapeutically useful antibiotics or antiviral agents
☐	☐	Enhance the virulence of a pathogen or render a nonpathogen virulent
☐	☐	Increase transmissibility of a pathogen
☐	☐	Alter the host range of a pathogen
☐	☐	Enable evasion of diagnostic/detection modalities
☐	☐	Enable the weaponization of a biological agent or toxin
☐	☐	Any other potentially harmful combination of experiments and agents

ChIP-seq

Data deposition

☒ Confirm that both raw and final processed data have been deposited in a public database such as GEO.

☒ Confirm that you have deposited or provided access to graph files (e.g. BED files) for the called peaks.

Data access links
May remain private before publication.

https://ddbj.nig.ac.jp/public/ddbj_database/gea/experiment/E-GEAD-000/

Files in database submission

293T-fanChIP-INPUT(T0226_IN) E-GEAD-320
293T-fanChIP-ENL rep1 (T0127_ENLx5) E-GEAD-402
293T-fanChIP-MOZ (T1117_MYST3) E-GEAD-322
293T-fanChIP-DOT1L (T0713_DOT1L) E-GEAD-320
293T-fanChIP-AF4 (T1117_AF4) E-GEAD-320
293T-fanChIP-RNAP2 non-P (T0329-parent) E-GEAD-402
293T-fanChIP-RNAP2 Ser5-P(T0226_RNAP2 Ser5-P) E-GEAD-320
293T-fanChIP-ENL rep 2 (T0226_ENL)E-GEAD-487
P31-fanChIP-INPUT(P0224_IN) E-GEAD-487
P31-fanChIP-ENL(P0217_ENL) E-GEAD-487
P31-fanChIP-RNAP2 Ser5-P(P0217_RNAP2 Ser5-P) E-GEAD-487
293Tpa-fanChIP-IN(T0725_Tpa_IN) E-GEAD-585
293TdMOZ-fanChIP-IN(T0715_TdMOZ_IN) E-GEAD-585
293Tpa-fanChIP-ENL(T0725_Tpa_ENL) E-GEAD-585
293TdMOZ-fanChIP-ENL(T0715_TdMOZ_ENL) E-GEAD-585
293Tpa-fanChIP-DOT1L(T0809_Tpa_DOT1L) E-GEAD-585
293TdMOZ-fanChIP-DOT1L(T00809_TdMOZ_DOT1L) E-GEAD-585

Genome browser session
(e.g. UCSC)

https://genome.ucsc.edu/s/akkanai%40edu.k.u%2Dtokyo.ac.jp/Fig2_hg19
https://genome.ucsc.edu/s/akkanai%40edu.k.u%2Dtokyo.ac.jp/Fig7_hg19

Methodology

Replicates

ChIP-seq analysis was performed once for most of the samples, and its reproducibility was confirmed by ChIP-qPCR analysis on selected targets. ChIP-seq analysis for ENL in HEK293T was performed for two replicates.

Sequencing depth

Sample name, Total number of reads, Uniquely mapped reads, Length of reads, Paired or single-end
293T-fanChIP-INPUT(T0713_INPUT), 35272474, 29469447, 50, single-end
293T-fanChIP-ENL rep1 (T0127_ENLx5), 29649601, 24606561, 50, single-end
293T-fanChIP-MOZ (T1117_MYST3), 28605527, 19713471, 50, single-end
293T-fanChIP-DOT1L (T0713_DOT1L), 27456969, 21862431, 50, single-end
293T-fanChIP-AF4 (T1117_AF4), 26394946, 20998044, 50, single-end
293T-fanChIP-RNAP2 non-P (T0329-parent), 36140826, 31175502, 50, single-end
293T-fanChIP-HBO1, 30383827, 25555603, 50, single-end
293T-fanChIP-INPUT(T0226_IN), 29574583, 25285507, 50, single-end
293T-fanChIP-RNAP2 Ser5-P(T0226_RNAP2 Ser5-P), 31647505, 27719450, 50, single-end
293T-fanChIP-ENL rep2 (T0226_ENL), 26126359, 22665033, 50, single-end
P31-fanChIP-INPUT(P0224_IN), 36894157, 30576899, 50, single-end
P31-fanChIP-ENL(P0217_ENL), 33437997, 27886822, 50, single-end
P31-fanChIP-RNAP2 Ser5-P(P0217_RNAP2 Ser5-P), 29621613, 25905364, 50, single-end

293Tpa-fanChIP-IN(T0725_Tpa_IN) 73,498,757 56,378,459 100 single-end
293TdMOZ-fanChIP-IN(T0715_TdMOZ_IN) 82,539,761 63,350,072 100 single-end
293Tpa-fanChIP-ENL(T0725_Tpa_ENL) 97,403,255 71,891,747 100 single-end
293TdMOZ-fanChIP-ENL(T0715_TdMOZ_ENL) 57,927,938 41,496,643 100 single-end
293Tpa-fanChIP-DOT1L(T0809_Tpa_DOT1L) 54,216,955 40,337,114 100 single-end
293TdMOZ-fanChIP-DOT1L(T00809_TdMOZ_DOT1L) 79,634,741 58,483,177 100 single-end

Antibodies

FLAG (M2) [ChIP: 1 µg/ 400 µL] Sigma-Aldrich Cat#:F3165; RRID:AB_259529
DOT1L [ChIP: 1 µL/400 µL] Bethyl Laboratories Cat#A300-953A; RRID: AB_805775
MOZ [ChIP: 1 µL/400 µL] Active motif Cat#39868; Discontinued
MOZ [ChIP: 1 µL/400 µL] Cell Signaling Technology Cat#78462
CyclinT1 [ChIP: 1 µg/400 µL] Santa cruz Biotechnology Cat#sc-8127; RRID: AB_2073892
ENL [ChIP:5 µg/400 µL] Cell Signaling Technology Cat#:14893S
RNAP2 Ser5-P [ChIP: 1 µg/400 µL] Millipore Cat#05-623; RRID: AB_309852
RNAP2 non-P [ChIP: 1 µg/400 µL] Abcam Cat#ab817; RRID: AB_306327
AF4 [ChIP: 1 µg/400 µL] Santa cruz Biotechnology Cat#sc-49350; RRID: AB_2226113
HBO1 [ChIP: 1 µg/400 µL] Abcam Cat#:70183; RRID:AB_1269226
ING4 [ChIP: 1 µg/400 µL] Abcam Cat#:108621; RRID:AB10860023

Peak calling parameters

Peak calling was not performed in this study.

Data quality

All sequenced reads that cleared the illumina's pass filter were mapped to human genome assembly hg19 using BWA 0.7.5.

Software

The alignment tags were counted and ppm was calculated every 25 bp from TSS and the ChIP signal distribution was plotted using NGSplot 2.61. The BAM alignment was converted to the bBigWig coverage files using bam2wig 1.6. and wigToBigWig.

Flow Cytometry

Plots

Confirm that:

- [x] The axis labels state the marker and fluorochrome used (e.g. CD4-FITC).
- [x] The axis scales are clearly visible. Include numbers along axes only for bottom left plot of group (a 'group' is an analysis of identical markers).
- [x] All plots are contour plots with outliers or pseudocolor plots.
- [ ] A numerical value for number of cells or percentage (with statistics) is provided.

Methodology

Sample preparation

For antibody staining, cells were incubated with antibodies at the concentrations recommended by the manufacturer for 30min, washed with PBS 3%FBS twice, and analyzed by FACS MELODY (BD).
For GFP detection, cells were re-suspendend in PBS 3%FBS, and analyzed by FACS MELODY (BD).

Instrument

BD FACS MELODY sorter (BD Biosciences)

Software

FlowJo (ver10.6.0, Tree Star)

Cell population abundance

N/A

Gating strategy

Alive cells were gated by the FSC/SSC gates as the starting population as shown in the supplementary information.

- [x] Tick this box to confirm that a figure exemplifying the gating strategy is provided in the Supplementary Information.

Magnetic resonance imaging

Experimental design

Design type

Indicate task or resting state; event-related or block design.

Design specifications

Specify the number of blocks, trials or experimental units per session and/or subject, and specify the length of each trial or block (if trials are blocked) and interval between trials.

Behavioral performance measures

State number and/or type of variables recorded (e.g. correct button press, response time) and what statistics were used to establish that the subjects were performing the task as expected (e.g. mean, range, and/or standard deviation across subjects).

Acquisition

Imaging type(s) — *Specify: functional, structural, diffusion, perfusion.*

Field strength — *Specify in Tesla*

Sequence & imaging parameters — *Specify the pulse sequence type (gradient echo, spin echo, etc.), imaging type (EPI, spiral, etc.), field of view, matrix size, slice thickness, orientation and TE/TR/flip angle.*

Area of acquisition — *State whether a whole brain scan was used OR define the area of acquisition, describing how the region was determined.*

Diffusion MRI ☐ Used ☐ Not used

Preprocessing

Preprocessing software — *Provide detail on software version and revision number and on specific parameters (model/functions, brain extraction, segmentation, smoothing kernel size, etc.).*

Normalization — *If data were normalized/standardized, describe the approach(es): specify linear or non-linear and define image types used for transformation OR indicate that data were not normalized and explain rationale for lack of normalization.*

Normalization template — *Describe the template used for normalization/transformation, specifying subject space or group standardized space (e.g. original Talairach, MNI305, ICBM152) OR indicate that the data were not normalized.*

Noise and artifact removal — *Describe your procedure(s) for artifact and structured noise removal, specifying motion parameters, tissue signals and physiological signals (heart rate, respiration).*

Volume censoring — *Define your software and/or method and criteria for volume censoring, and state the extent of such censoring.*

Statistical modeling & inference

Model type and settings — *Specify type (mass univariate, multivariate, RSA, predictive, etc.) and describe essential details of the model at the first and second levels (e.g. fixed, random or mixed effects; drift or auto-correlation).*

Effect(s) tested — *Define precise effect in terms of the task or stimulus conditions instead of psychological concepts and indicate whether ANOVA or factorial designs were used.*

Specify type of analysis: ☐ Whole brain ☐ ROI-based ☐ Both

Statistic type for inference (See Eklund et al. 2016) — *Specify voxel-wise or cluster-wise and report all relevant parameters for cluster-wise methods.*

Correction — *Describe the type of correction and how it is obtained for multiple comparisons (e.g. FWE, FDR, permutation or Monte Carlo).*

Models & analysis

n/a	Involved in the study
☐	☐ Functional and/or effective connectivity
☐	☐ Graph analysis
☐	☐ Multivariate modeling or predictive analysis

Functional and/or effective connectivity — *Report the measures of dependence used and the model details (e.g. Pearson correlation, partial correlation, mutual information).*

Graph analysis — *Report the dependent variable and connectivity measure, specifying weighted graph or binarized graph, subject- or group-level, and the global and/or node summaries used (e.g. clustering coefficient, efficiency, etc.).*

Multivariate modeling and predictive analysis — *Specify independent variables, features extraction and dimension reduction, model, training and evaluation metrics.*
